# *Wolbachia* infection and genetic diversity of Italian populations of *Philaenus spumarius*, the main vector of *Xylella fastidiosa* in Europe

**DOI:** 10.1371/journal.pone.0272028

**Published:** 2022-08-29

**Authors:** Giorgio Formisano, Luigi Iodice, Pasquale Cascone, Adriana Sacco, Roberta Quarto, Vincenzo Cavalieri, Domenico Bosco, Emilio Guerrieri, Massimo Giorgini

**Affiliations:** 1 Istituto per la Protezione Sostenibile delle Piante, Consiglio Nazionale delle Ricerche, Portici, Italy; 2 Istituto per la Protezione Sostenibile delle Piante, Consiglio Nazionale delle Ricerche, Bari, Italy; 3 Dipartimento di Scienze Agrarie, Forestali e Alimentari, Università degli Studi di Torino, Grugliasco, Italy; Lund University, SWEDEN

## Abstract

*Philaenus spumarius* is a cosmopolitan species that has become a major threat to European agriculture being recognized as the main vector of the introduced plant pathogen *Xylella fastidiosa*, the agent of the “olive quick decline syndrome”, a disease which is devastating olive orchards in southern Italy. *Wolbachia* are bacterial symbionts of many insects, frequently as reproductive parasites, sometime by establishing mutualistic relationships, able to spread within host populations. *Philaenus spumarius* harbors *Wolbachia*, but the role played by this symbiont is unknown and data on the infection prevalence within host populations are limited. Here, the *Wolbachia* infection rate was analyzed in relation to the geographic distribution and the genetic diversity of the Italian populations of *P*. *spumarius*. Analysis of the *COI* gene sequences revealed a geographically structured distribution of the three main mitochondrial lineages of *P*. *spumarius*. *Wolbachia* was detected in half of the populations sampled in northern Italy where most individuals belonged to the western-Mediterranean lineage. All populations sampled in southern and central Italy, where the individuals of the eastern-Mediterranean lineage were largely prevalent, were uninfected. Individuals of the north-eastern lineage were found only in populations from the Alps in the northernmost part of Italy, at high altitudes. In this area, *Wolbachia* infection reached the highest prevalence, with no difference between north-eastern and western-Mediterranean lineage. Analysis of molecular diversity of *COI* sequences suggested no significant effect of *Wolbachia* on population genetics of *P*. *spumarius*. Using the MLST approach, six new *Wolbachia* sequence types were identified. Using FISH, *Wolbachia* were observed within the host’s reproductive tissues and salivary glands. Results obtained led us to discuss the role of *Wolbachia* in *P*. *spumarius*, the factors influencing the geographic distribution of the infection, and the exploitation of *Wolbachia* for the control of the vector insect to reduce the spread of *X*. *fastidiosa*.

## Introduction

*Wolbachia* Hertig (α-Proteobacteria) are maternally-inherited intracellular bacteria that establish symbiotic relationships with a wide range of arthropods and nematodes, including 40–60% of the insect species [1, 2]. Most of the *Wolbachia* strains are known to be reproductive manipulators of their hosts and to increase their prevalence within host populations by improving the reproductive fitness of the infected females [3]. Cytoplasmic incompatibility (CI) is the most frequent reproductive manipulation used by *Wolbachia* to spread within host populations. CI is a form of sterility that occurs when infected males mate with uninfected females (unidirectional CI) or when mates harbor different *Wolbachia* strains (bidirectional CI), resulting in the death of the progeny during early embryogenesis [3]. Conversely, viable and fertile progeny is produced by infected females when mated with infected or uninfected males. The result of CI is a reduction of the reproduction of uninfected females. Consequently, the prevalence of *Wolbachia* can increase within the host population and under certain conditions can rapidly reach a stable equilibrium, even close to 100% [4, 5]. *Wolbachia* can also spread within host populations by inducing female-biased sex ratios of host progeny through parthenogenesis, feminization or male-killing [3, 6, 7]. Other strains of *Wolbachia* are beneficial for their host, establishing obligate [8, 9] or facultative mutualistic relationships by increasing host fecundity [10, 11], conferring host protection against pathogen infections [12–15] and mediating host plant specialization [16].

The ability of *Wolbachia* to interfere with host reproduction and to be pervasive within insect populations makes this endosymbiont promising for the development of innovative methods for biological control of insect pests and to reduce the spread of vector-borne pathogens [17–20]. To date, most of the *Wolbachia*-based control methods rely on CI and artificial transfection of the target insects with a *Wolbachia* strain, they do not naturally carry, expressing a strong CI phenotype. By the Incompatible Insect Technique (IIT) [21, 22], CI *Wolbachia*-infected males are repeatedly distributed in large numbers in the field to determine the suppression of a pest. Because wild females will produce non-viable embryos when mated by infected males, the inundative release of incompatible males, that outcompete wild males in mating wild females, will reduce the size of the target population. Alternatively to IIT, the population replacement approach uses CI to favor the spread of a *Wolbachia* strain with a pathogen-blocking phenotype (inhibition of pathogen infection, replication and transmission) [23]. This strategy is based on the release of both males and females infected by *Wolbachia* and on the reproductive advantage that CI provides to infected females. Infected offspring, if able to settle permanently, will lead over time to a progressive reduction in the frequency of the uninfected insect population with the consequent reduction of the vector competence and decline of the transmitted pathogen in the treated area [22–24]. Up to now, these strategies have been successfully applied for protecting humans against mosquitos and vectored pathogens (e.g. arboviruses). Recently, the brown planthopper, *Nilaparvata lugens* (Stål), was transfected with a *Wolbachia* strains which, in addition to expressing strong CI, inhibits the transmission of a virus that severely damages rice crops, thus attenuating the intensity of the plant disease [25]. This study paved the way for the application of the *Wolbachia*-based approach to the control of agricultural pests and the pathogens they transmit [26]. Other potential approaches for vector and disease control aim to exploit CI-*Wolbachia* to introduce transgenes into the target population [27, 28] but are still under study and far from being applied in the field [22, 29, 30].

The spittlebug *Philaenus spumarius* L. (Hemiptera: Aphrophoridae) is a xylem sap-sucking insect whose nymphs develop in meadows, feeding on a wide range of host plants, mainly dicots. Adults can remain on the herbaceous vegetation but often disperse onto woody hosts during summer. This very polyphagous species can also feed on crops but has reached the pest status very rarely [31]. Recently, after the introduction of the plant pathogenic bacterium *Xylella fastidiosa* Wells *et al*. (γ-Proteobacteria) in southern Europe, *P*. *spumarius* has become one of the major threat to European agriculture [32, 33], due to its ability to transmit *X*. *fastidiosa* coupled to a wide distribution throughout the Continent [31, 34–37]. *Xylella fastidiosa* is now present in Italy, Spain, France and Portugal and has been eradicated in Germany and Switzerland [38]. In the Puglia region of Italy, *P*. *spumarius* is the main vector of *X*. *fastidiosa* subspecies *pauca*, the causal agent of the “olive quick decline syndrome” (OQDS). OQDS disease is quickly devastating the olive growing in Puglia with significant economic, environmental and social impacts [39, 40]. In Spain, *P*. *spumarius* is a vector of *X*. *fastidiosa* subspecies *fastidiosa* to grapevine in Mallorca [41] and of the almond leaf scorch in Alicante, caused by both *multiplex* and *fastidiosa* subspecies [33]. The distribution of the bacterium is expected to increase in the Mediterranean Basin with a severe impact in olive, grape and fruit producing areas [32, 42, 43].

*Philaenus spumarius* is a cosmopolitan species originated in the Palearctic region and distributed throughout the Holarctic region [31, 44]. According to most recent phylogeographic studies based on mitochondrial and nuclear genes, this species differentiates in two main genetic groups, namely the north-eastern (NE) and the south-western (SW) lineages, with the latter divided into two sub-groups, namely the eastern-Mediterranean and the western-Mediterranean lineages [45–47]. This phylogeographic structure was also largely supported by genomic data [48]. The NE lineage is distributed in the Anatolian and Caucasian regions, Central and Northern Europe, and eastern Asia, and has been introduced in North America and New Zealand. The distribution of the SW lineage is overall more southerly but with differences between its two sub-groups. The western-Mediterranean lineage is mainly distributed in the Iberian Peninsula, but is present in the entire Mediterranean region and some other areas of Western Europe and Middle East, and has been introduced into North America. The eastern-Mediterranean lineage is distributed in the Balkans, Mediterranean region, Eastern Europe and Middle East. The NE and SW lineages are geographically separated in most part of the natural range of *P*. *spumarius*, with contact zones occurring in some region [46, 49]. *Philaenus spumarius* feeds on xylem sap, a fluid with low amino acid and sugar content. To overcome this nutritional deficiency, it relies on the complementary biosynthetic pathways of two obligate bacterial symbionts [50, 51], each one compartmentalized in specialized host cells (bacteriocites) of different bacteriomes [52]. *Philaenus spumarius* can also harbors facultative bacterial symbionts, namely *Arsenophonus*, *Cardinium*, *Rickettsia* and *Wolbachia*, whose role in spittlebug populations is not yet known [54]. *Wolbachia* were detected in *P*. *spumarius* populations of different geographic areas [53–55] and the infection prevalence was shown to differ between mitochondrial lineages of *P*. *spumarius* and between the regions of the geographic range [53]. In the latter study, *Wolbachia* was considered as a possible factor of reproductive isolation, via cytoplasmic incompatibility, between populations of NE and SW lineage in the Carpathians contact zone. However, data on the prevalence of *Wolbachia* infection within the host populations are limited since very few individuals per population were analyzed by [53]. A recent study from Greece, indicated low infection rates in populations of the eastern-Mediterranean lineage [54].

Despite the great importance of *P*. *spumarius* in Italy as vector of *X*. *fastidiosa*, there is only little information on the genetic diversity of the Italian populations and the prevalence of *Wolbachia* infections. The few data available originate from two studies on the global diversity of *P*. *spumarius* and suggest that the Italian populations are largely composed of individuals of the SW lineage. However, a clear geographic structure cannot be inferred from these papers as only a few dozen individuals from Italy were analyzed [45, 46]. Similarly, the extremely low numbers of individuals screened for *Wolbachia* infection [53] do not allow a deep understanding of the actual infection prevalence among the Italian populations of *P*. *spumarius*. Here, we aimed to broaden knowledge about *Wolbachia* ecology in the populations of *P*. *spumarius* of Italy. Understanding the factors influencing the ecology of *Wolbachia* is of interest to evaluate the applicability of *Wolbachia*-based strategies to control vector populations and to contrast the spread of *X*. *fastidiosa*. Therefore, in order to highlight the factors that may affect the distribution of *Wolbachia* among the populations of *P*. *spumarius* across Italy, the main aims of this work were to: i) study the prevalence of *Wolbachia* infection in relation to the geographical distribution and mitochondrial genetic diversity of *P*. *spumarius*; ii) characterize *Wolbachia* strains and their association with the host mitochondrial genotype; iii) understand the possible impact of *Wolbachia* infection on the population genetics of *P*. *spumarius*; v) enhance the knowledge on the symbiotic interaction between *Wolbachia* and *P*. *spumarius* through the study of the symbiont tissue tropism.

## Materials and methods

### Insect collection

*Philaenus spumarius* populations were sampled from 2015 to 2019 in 75 locations from southern to northern Italy ranging from sea level to 1878 m altitude. Overall, 52 populations were sampled in four regions of southern Italy, including the Puglia region, the focal area of *X*. *fastidiosa* epidemics. Four populations were sampled in a region of central Italy and 19 populations in four regions of northern Italy (S1 Table). *Philaenus spumarius* adults were collected, from spring to autumn, using a sweep net and an entomological aspirator in meadows, natural vegetation surrounding crops or on cultivated fruit trees (S1 Table). Immediately after collection, insects were stored in absolute ethanol at -20°C before DNA extraction. Because the only other species of *Philaenus* recorded from Italy is *Philaenus italosignus* Drosopoulos & Remane, which is sometime found in sympatry with *P*. *spumarius* in south-central Italy [56–58], before the individuals were grinded for DNA extraction, their species identity was confirmed at a stereoscope by examining the body size and the external morphology of male terminalia [59]. In addition, 1–2 males (not used for DNA extraction) for population (not all populations sampled) were identified at a microscope by examining the morphology of the aedeagus [59–61]. All individuals were assigned to *P*. *spumarius*.

### DNA extraction

Single individuals were dissected at a stereoscope to remove the wings and separate the abdomen and the head from the thorax. Genomic DNA was extracted from abdomen and, if PCR revealed the presence of *Wolbachia*, from the head. Insect abdomen or head was grinded with a pestle in a 1.5 ml centrifuge tube using a Chelex-proteinase K protocol [62]. The supernatant containing the DNA was removed after centrifugation and stored at –20°C. DNA extracted from the head was cleaned with the ISOLATE II Plant DNA kit (Bioline Meridian Life Science Inc., U.S.A) before running PCR reactions to improve the quality of the amplification products.

### *Philaenus spumarius* molecular typing and phylogeographic structure analysis

The *cytochrome oxidase subunit I* (*COI*) gene of single individuals was amplified using the DNA extracted from the insect’s abdomen and the primer combination C1-J-2195 and TL2-N-3014 [63]. Reactions were performed in 15 μl volumes containing 3 μl of 5X GoTaq buffer (Promega, Madison, Wisconsin, USA), 0.5 μl dNTPs (2.5 mM each), 0.75 μl of forward and reverse primer (10 μM each), 0.04 μl GoTaq G2 DNA Polymerase (Promega, Madison, Wisconsin, USA) (5 u/μl), and 2 μl template DNA. Amplifications were achieved using a Mycycler thermocycler (Bio-Rad, Hercules, California, USA) programmed at 1 min at 94°C, followed by 40 cycles of 30 s at 94°C, 90 s at 48°C, and 60 s at 72°C, and a final step of 7 min at 72°C. PCR products were visualized after electrophoresis on 1% agarose gel stained with ethidium bromide (Promega, Madison, Wisconsin, USA) to confirm the amplification. Fragments obtained were sequenced in both sense and antisense directions by adopting standard service (Macrogen Inc., Seoul, South Korea and Microsynth Seqlab, Balgach, Switzerland). The chromatograms obtained were viewed and edited in Chromas v.2.6.4 (Technelysium, South Brisbane, Queensland, Australia). No double peaks, which may indicate heteroplasmy in *P*. *spumarius* [49], were detected. Protein-coding was checked by translating the sequences into amino acids. A region of 539 bp within the sequenced fragment was used for subsequent analyses [46]. Overall, 265 individuals of *P*. *spumarius* sampled throughout Italy were sequenced including 117 from southern Italy, 10 from central Italy and 138 from northern Italy (34, 2 and 19 populations sequenced, respectively) (S1 Table). Haplotypes were designated following the progressive numbering by [46] and additional new haplotypes identified in our study were numbered starting from H72. *Philaenus spumarius* sequences were deposited in GenBank under accession numbers MN812359-MN812427 and MW737706-MW737787) (S2 Table).

**Fig 1:**
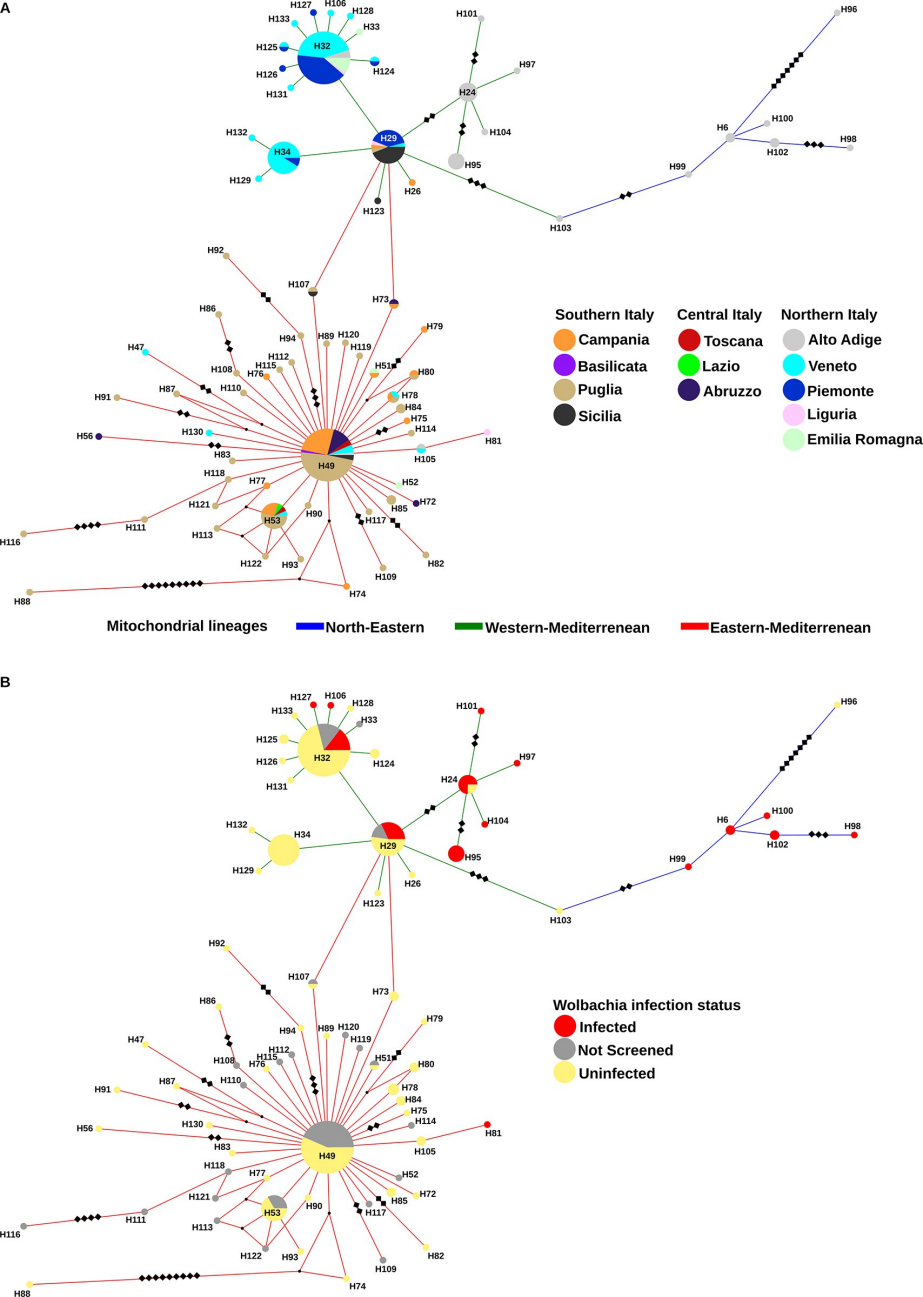
Median joining haplotype network obtained from *COI* gene sequences of *Philaenus spumarius* collected from different regions of Italy. (A) Haplotype frequency by geographic region. (B) Haplotype frequency by *Wolbachia* infection. Circle sizes are proportional to haplotype frequency. Colors correspond to the region of origin of the haplotype (A) and the *Wolbachia* infection status (B). Small black dot vertices represent missing or unsampled haplotypes. Diamonds on branches represent the number of mutations. No diamond on branches means one mutation.

The phylogeographic structure of *P*. *spumarius* populations was studied by a median-joining haplotype network following [46]. A matrix was assembled using 265 sequences of the Italian specimens produced in this study and the sequences by [46], these latter including 159 sequences from Europe, among which 17 from Italy, and 31 from other continents. *COI* sequences were aligned using the Muscle alignment tool in Aliview 1.26 [64] and were trimmed to 539 bp. The online service FaBox DNA Collapser [65] was used to reduce the sequence dataset to haplotypes and to create input files for subsequent analyses. Median-joining phylogenetic networks [66] were generated to infer relationships among haplotypes using PopART [67] and visualized using Cytoscape [68]. Two networks were produced. In the first one, haplotypes were visualized according to the marco-regions of origin (sensu [46]) (S1 Fig) and Italy was divided into southern, central and northern Italy. The other network included only the Italian haplotypes grouped according to the regions of origin (Campania, Puglia, Basilicata and Sicilia, for southern Italy; Abruzzo, Lazio and Toscana, for central Italy; Alto Adige, Emilia Romagna, Liguria, Piemonte and Veneto, for northern Italy) (Fig 1A). Genetic divergence (uncorrected *p*-distance, that is the number of base differences per site) between and within mitochondrial phylogeographic lineages was calculated using MEGA X [69].

### Screening for *Wolbachia* infection

The prevalence of *Wolbachia* infection was investigated in *P*. *spumarius* populations sampled in 37 locations in south-central Italy and 17 locations in northern Italy (Table 1 and S1 Table). Overall, 783 adults were screened by PCR using DNA extracted from abdomen and the *Wolbachia* specific primers ftsZ_F1 and ftsZ_R1, amplifying a region of the *ftsZ* gene [70]. Negative PCRs were confirmed with the *Wolbachia* specific primers Wol-16S-F and Wol-16S-R [71] targeting the 16S rRNA gene. DNA quality was checked by amplifying a region of the insect mitochondrial *COI* gene (see above) and the 16S rRNA gene of the primary endosymbiont *Sulcia mullerii* using the primers 10_CFB_FF and 1515_R [72]. *Wolbachia* screening was also run on DNA extracted from the heads of infected individuals of five populations (Table 2). Infection rates were compared between populations and sexes by the G-test of independence or when the G-test was not applicable, by the Fisher’s exact test of independence. Both tests were run using the spreadsheets available on line in [73].

**Table 1:** *Wolbachia* infection in Italian populations of *Philaenus spumarius*.

Region	Population	Location^†^	Coordinates	Altitude (m asl)	N. of individuals screened for *Wolbachia* (infection rate)		
					Females	Males	Total
**NORTHERN ITALY**							
**Alto Adige**	Aa1	Compaccio	46.541156, 11.617049	1848	36 (75%)	11 (81.8%)	47 (76.6%)
	Aa2	Castelrotto	46.564172, 11.551320	1000	38 (36.8%)	20 (10%)	58 (27.6%)
	Aa3	San Michele	46.578300, 11.602030	1283	24 (83.3%)	16 (75%)	40 (80%)
	*Total*		* *	* *	*98 (62*.*2%)*	*47 (48*.*9%)*	*145 (57*.*9%)*
**Piemonte**	Pi1	Asti	44.92192, 8.1957	185	10 (60%)	5 (40%)	15 (53.3%)
	Pi3	Venaria	45.147702, 7.597928	290	9 (33.3%)	4 (100%)	13 (53.8%)
	Pi5	Castellero	44.915803, 8.067924	200	3 (0%)	4 (0%)	7 (0%)
	Pi6	Castellamonte	45391126, 7.683194	379	5 (20%)	1 (0%)	6 (16.7%)
	Pi7	Fossano	44.532195, 7.745778	350	2 (0%)	3 (0%)	5 (0%)
	*Total*				*29 (34*.*5%)*	*17 (35*.*3%)*	*46 (34*.*8%)*
**Veneto**	Ve1	Thiene	45.677533, 11.511060	89	5 (0%)	0 (-)	5 (0%)
	Ve3	Barbarano V.	45.409000, 11.546725	35	5 (0%)	6 (0%)	11 (0%)
	Ve4	Bardolino	45.541489, 10.732594	115	5 (0%)	6 (33.3%)	11 (18.2%)
	Ve5	Belvedere	45.685838, 11.771236	70	5 (20%)	0 (-)	5 (20%)
	Ve6	Lazise	45.508711, 10.738042	94	2 (0%)	9 (11.1%)	11 (9.1%)
	Ve7	Montegrotto T.	45.315586, 11.772342	114	2 (0%)	4 (0%)	6 (0%)
	Ve8	Montecchio P.	45.654492, 11.557116	70	5 (20%)	0 (-)	5 (20%)
	Ve9	Bussolengo	45.447969, 10.862103	105	6 (0%)	7 (0%)	13 (0%)
	*Total*				*35 (5*.*7%)*	*32 (9*.*4%)*	*67 (7*.*5%)*
**Liguria**	Li1	Finale Ligure	44.18089, 8.363513	257	3 (33.3%)	1 (0%)	4 (25%)
**SOUTH-CENTRAL ITALY**							
**Abruzzo**	Ab1	Colonnella	42.867961, 13.848218	190	11 (0%)	2 (0%)	13 (0%)
	Ab2	Torino di Sangro	42.205420, 14.535794	65	10 (0%)	2 (0%)	12 (0%)
	Ab3	Pineto	42.605431, 14.041484	50	9 (0%)	2 (0%)	11 (0%)
	Ab4	Spoltore	42.227320, 14.423415	240	8 (0%)	5 (0%)	13 (0%)
	*Total*				*38 (0%)*	*11 (0%)*	*49 (0%)*
**Campania**	Ca1	Montesarchio	41.086186,14.654908	548	10 (0%)	7 (0%)	17 (0%)
	Ca2	Giffoni V.P.	40.753892, 14.921383	432	8 (0%)	10 (0%)	18 (0%)
	Ca3	Montevergine	40.937361, 14.718131	1431	8 (0%)	9 (0%)	17 (0%)
	Ca4	Vico Equense	40.65813, 14.46419	563	10 (0%)	10 (0%)	20 (0%)
	Ca5	Bellizzi	40.648889, 14.961389	81	10 (0%)	10 (0%)	20 (0%)
	Ca6	Giffoni V.P.	40.707881, 14.956458	205	10 (0%)	10 (0%)	20 (0%)
	Ca7	Giffoni V.P.	40.765158, 14.914103	509	0 (0%)	5 (0%)	5 (0%)
	Ca8	Giffoni V.P.	40.706744, 14.940078	153	15 (0%)	15 (0%)	30 (0%)
	Ca9	Castellamare di S.	40.680019, 14.49622	289	7 (0%)	7 (0%)	14 (0%)
	Ca10	Vico Equense	40.67025, 14.470085	1027	3 (0%)	2 (0%)	5 (0%)
	Ca11	Vico Equense	40.669731, 14.458779	729	1 (0%)	2 (0%)	3 (0%)
	Ca12	Vico Equense	40.664322, 14.435961	196	5 (0%)	8 (0%)	13 (0%)
	Ca13	Torre le Nocelle	41.028305, 14.961900	420	9 (0%)	9 (0%)	18 (0%)
	Ca14	Bocca della Selva	41.37605,14.51044	1313	18 (0%)	14 (0%)	32 (0%)
	Ca15	Montesarchio	41.071979,14.656936	397	12 (0%)	7 (0%)	19 (0%)
	Ca16	Tocco Caudio	41.103478,14.634976	676	10 (0%)	10 (0%)	20 (0%)
	Ca17	Frasso Telesino	41.139076,14.518679	309	8 (0%)	8 (0%)	16 (0%)
	Ca18	San Mango sul C.	40.958442, 14.970316	500	6 (0%)	4 (0%)	10 (0%)
	Ca19	San Martino V.C.	41.017556, 14.663333	366	13 (0%)	8 (0%)	21 (0%)
	Ca20	Pannarano	41.004758, 14.701219	406	9 (0%)	9 (0%)	18 (0%)
	Ca21	Pannarano	41.022758, 14.703211	277	4 (0%)	3 (0%)	7 (0%)
	Ca22	Portici	40.815175, 14.351439	91	4 (0%)	0 (0%)	4 (0%)
	*Total*				*180 (0%)*	*167 (0%)*	*347 (0%)*
**Puglia**	Pu1	Alliste	39.94775, 18.08637	52	12 (0%)	10 (0%)	22 (0%)
	Pu2	Gallipoli	40.05457, 17.99425	15	12 (0%)	12 (0%)	24 (0%)
	Pu3	Ruvo di Puglia	41.11695, 16.48882	230	4 (0%)	4 (0%)	8 (0%)
	Pu4	Fasano	40.84022, 17.36070	368	5 (0%)	5 (0%)	10 (0%)
	Pu7	Locorotondo	40.76916, 17.33388	365	8 (0%)	2 (0%)	10 (0%)
	Pu12	Galugnano	40.25643, 18.21200	18	2 (0%)	2 (0%)	4 (0%)
	Pu13	Leverano	40.30833, 17.95833	41	5 (0%)	5 (0%)	10 (0%)
	Pu16	Poggiardo	40.05262, 18.37901	87	5 (0%)	10 (0%)	15 (0%)
	Pu23	Latiano	40.60750, 17.71694	108	5 (0%)	5 (0%)	10 (0%)
	*Total*				*58 (0%)*	*55 (0%)*	*113 (0%)*
**Sicilia**	Si3	Ragusa	36.91868, 14.71028	554	6 (0%)	6 (0%)	12 (0%)

^†^See S1 Table for further details on the sampled populations.

**Table 2:** PCR detection of *Wolbachia* in the head of infected individuals of *Philaenus spumarius*. Percentage of infected heads calculated on the number of infected individuals.

Region	Population	Individuals infected/ Individuals screened for *Wolbachia*			% *Wolbachia*-infected heads		
		Females	Males	Total	Females	Males	Total
**Alto Adige**	Aa1	27/36	9/11	36/47	70.4%	33.3%	61.1%
	Aa2	14/38	2/20	16/58	71.4%	0%	62.5%
	Aa3	20/24	12/16	32/40	35%	16.7%	28.1%
		*61/98*	*23/47*	*84/145*	*59%*	*21*.*7%*	*48*.*8%*
**Piemonte**	Pi1	6/10	2/5	8/15	66.7%	100%	75%
	Pi3	3/9	4/4	7/13	66.7%	50%	57.1%
		*9/19*	*6/9*	*15/28*	*66*.*7%*	*66*.*7%*	*66*.*7%*
**Total**		70/117	29/56	99/173	60%	31%	52%

### Sex ratio in the populations of *Philaenus spumarius*

The sex ratio was assessed in five *Wolbachia*-infected populations of northern Italy (Alto Adige, Piemonte and Liguria) and 27 uninfected populations of southern Italy (Campania, Puglia and Sicilia) (S3 Table). The G-test of independence [73] was used to compare the sex ratio of *P*. *spumarius* populations within and between geographic regions.

### Diversity of *Philaenus spumarius* mtDNA

The Italian populations of *P*. *spumarius* screened for *Wolbachia* infection and with at least four individuals sequenced for the mitochondrial *COI* gene were considered for the analysis of molecular diversity. Overall, 27 populations and 226 individuals were analyzed (S1 Table). In detail, 16 populations were from three regions of northern Italy (Alto Adige, Piemonte and Veneto) and 11 populations from four regions of south-central Italy (Campania, Puglia and Sicilia in the south; Abruzzo in the center). Population genetic parameters of mtDNA, including number of haplotypes, haplotype diversity (*Hd*), number of segregating sites (*S*), nucleotide diversity (*π*) and mean number of pairwise differences (*k*) were calculated for *P*. *spumarius* populations grouped on the basis of the geographic region of origin (S4 Table). Additional analyses of molecular diversity were run by grouping the haplotypes according to their mitochondrial lineage (S5 Table) or infection status (infected and uninfected haplotypes) (S6 Table). Tajima’s D [74] and Fu’s Fs [75] statistics were computed to detect any departure from neutral evolution. Both neutrality test statistics are expected to be zero if sequences are evolving in a neutral way. Values significantly less than zero mean a higher-than-expected number of low frequency variants as a result of population expansion or recent selective sweep, as that associated to the invasion of maternally-inherited symbionts, e.g. *Wolbachia*. Because *Wolbachia* and mitochondria are simultaneously inherited through the egg cytoplasm, if the symbionts confer a reproductive advantage to the infected individuals over the uninfected ones, the spread of *Wolbachia* within the host populations will hitchhike the mitochondrial variants associated with the infection. Then, the uninfected mitochondrial variants will be swept from the infected host populations [76–78]. Under this circumstance, molecular diversity parameters and negative values of neutrality test statistics of a swept infected population are expected to be lower than those of an uninfected population. Finally, the analysis of molecular variance (AMOVA) was performed to assess the relationship between haplotype variations and possible causes of haplotype variability including geographic isolation and *Wolbachia* infection. The analysis was run on *P*. *spumarius* populations grouped by geographic origin or *Wolbachia* infection status (10 populations infected and 17 uninfected) (S7 Table). An additional AMOVA was conducted by grouping the individuals of different geographic areas according their infection status (S8 and S9 Tables). The software Arlequin 3.5.1.2 [79] was used to calculate the molecular diversity parameters and the neutrality test statistics, and to run the AMOVA.

### *Wolbachia* molecular typing and phylogenetic analysis

The strains of *Wolbachia* found in *P*. *spumarius* were characterized using the MLST approach suggested by [70] based on the amplification and sequencing of a region of the bacterial genes *gatB*, *coxA*, *hcpA*, *fbpA* and *ftsZ*, and subsequent assignation of an allelic profile, the so called sequence type (ST) (http://pubmlst.org/Wolbachia/). As an additional molecular marker, the *wsp* gene was amplified and sequenced and each *Wolbachia* strain was typed based on the amino acid sequence of the hypervariable regions of the WSP protein. Primers and PCR cycling conditions were as suggested by [70], while PCR reactions were performed as reported above for the *COI* gene. Overall, 20 infected individuals with known mitochondrial haplotype were processed for molecular typing of *Wolbachia* (Table 3). *Wolbachia* gene sequences were deposited in GenBank under accession numbers MN812292-MN812358 (S10 Table). Alignments of single and concatenated (2079 bp) MLST gene sequences of *Wolbachia* STs harbored by Italian *P*. *spumarius* were examined in Mesquite 3.61 [80] and verified by eye for errors. The data set was implemented with reference STs of most *Wolbachia* supergroups retrieved from the MLST database. In addition, we included in the alignment the sequences of *Wolbachia* endosymbionts of *P*. *spumarius* from [53]; these sequences were not available in the *Wolbachia* MLST database (no sequence type formally defined for *P*. *spumarius*) and were retrieved from GenBank to be included in our analysis. Similarly, alignment of the *wsp* gene sequences was also examined, although other *wsp* gene sequences of *P*. *spumarius* were not available in both GenBank and MLST database.

**Table 3:** *Wolbachia* sequence types (ST) in *Philaenus spumarius* and their association with host population and haplotype. New STs and allelic variants in the MLST database are in italic.

Population	Region	Host COI haplotype	Mt lineage^†^	*Wolbachia* ST	MSLT allelic profile					*wsp* gene	WSP HVR profile
					*gatB*	*coxA*	*hcpA*	*ftsZ*	*fbpA*		(HVR1-HVR4)
**Aa1**	Alto Adige	H6	NE	*546*	150	134	227	124	*470*	698	201-35-24-84
**Aa1**	Alto Adige	H98	NE	*545*	150	134	141	124	*470*	698	201-35-24-84
**Aa1**	Alto Adige	H6	NE	*539*	150	134	159	124	*470*	698	201-35-24-84
**Aa1**	Alto Adige	H102	NE	*537*	135	120	159	108	197	-	n.d.-18-16-23
**Aa2**	Alto Adige	H95	W-Med	*538*	150	*301*	159	124	*470*	698	201-35-24-84
**Aa2**	Alto Adige	H97	W-Med	*539*	150	134	159	124	*470*	698	201-35-24-84
**Aa2**	Alto Adige	H95	W-Med	*538*	150	*301*	159	124	*470*	698	201-35-24-84
**Aa2**	Alto Adige	H101	W-Med	*539*	150	134	159	124	*470*	698	201-35-24-84
**Aa3**	Alto Adige	H24	W-Med	*539*	150	134	159	124	*470*	698	201-35-24-84
**Aa3**	Alto Adige	H99	NE	*538*	150	*301*	159	124	*470*	698	201-35-24-84
**Aa3**	Alto Adige	H24	W-Med	*539*	150	134	159	124	*470*	698	201-35-24-84
**Aa3**	Alto Adige	H95	W-Med	*538*	150	*301*	159	124	*470*	698	201-35-24-84
**Aa3**	Alto Adige	H100	NE	*539*	150	134	159	124	*470*	698	201-35-24-84
**Aa3**	Alto Adige	H24	W-Med	*539*	150	134	159	124	*470*	698	201-35-24-84
**Aa3**	Alto Adige	H104	W-Med	*539*	150	134	159	124	*470*	698	201-35-24-84
**Li1**	Liguria	H81	E-Med	*549*	150	*302*	159	124	*470*	698	201-35-24-84
**Pi1**	Piemonte	H29	W-Med	*539*	150	134	159	124	*470*	698	201-35-24-84
**Pi1**	Piemonte	H32	W-Med	*549*	150	*302*	159	124	*470*	698	201-35-24-84
**Pi6**	Piemonte	H32	W-Med	*549*	150	*302*	159	124	*470*	698	201-35-24-84
**Ve8**	Veneto	H106	W-Med	*549*	150	*302*	159	124	*470*	698	201-35-24-84

^**†**^ Mt lineage = mitochondrial lineage; E-Med = eastern-Mediterranean lineage; NE = north-eastern lineage; W-Med = western-Mediterranean lineage.

The phylogenetic analysis was performed by Bayesian inference (BI) and the concatenated matrix was exported from Mesquite to Mr. Bayes 3.2.6 [81]. Substitution models were selected by PartitionFinder 2.1.1 [82], based on the AICc criterion. As for the MLST genes, BI was conducted according to the best partitioning scheme selected by PartitionFinder using ‘all’ search algorithm with branch lengths linked. The BI tree was obtained by implementing the substitution model GTR+I for gene partitions *gatB*+*fbpA*, *coxA*, *hcpA* and *ftsZ*. *Wolbachia* ST-402 (supergroup M) hosted by the aphid *Pentalonia nigronervosa* Coquerel was used as outgroup. For the *wsp* gene, the BI tree was obtained by implementing the substitution model GRT+I. The allele *wsp*-34 of *Wolbachia* ST-37 (supergroup D) hosted by the nematode *Brugia malayi* Brug was used as outgroup. Two runs of four Monte Carlo Markov chains (3 “heated” and 1 “cold”) were run in parallel in MrBayes for 15,000,000 generations for concatenated MLST genes and 5,000,000 generations for *wsp* gene. Trees were sampled every 1,000 generations. Convergence of the separate runs was checked using the average deviation of split frequencies diagnostic and the PSRF parameter (close to 1.00 for all parameters). The burn-in value was set at 25% of sampled topologies. Trees were imported into the tree editor TreeGraph 2.14.0–771 beta [83] for annotation and layout. In addition to BI inference, *Wolbachia* phylogeny based on concatenated MLST gene sequences was constructed using maximum likelihood (ML) analysis performed in MEGA X [54]. ML tree was obtained implementing the T92+G+I substitution model, selected in MEGA X as the better to describe the substitution pattern (lowest BIC score), and five discrete gamma categories. Missing data were treated with the pairwise-deletion option and all codon positions were included in the analysis. For tree inference, the nearest-neighbor-interchange (NNI) heuristic method was used and the initial tree selected using the NJ/BioNJ option. ML branch support was based on 1,000 bootstrap replications. Genetic divergence (uncorrected p-distance) between *Wolbachia* STs was calculated using MEGA X [69]. Because MLST genes undergo to recombination events between Wolbachia strains [84–87], evolutionary relationships of *Wolbachia* can be difficult to interpret using standard phylogenetic analyses [88, 89]. To account for potentially conflicting signals due to recombination, concatenated MLST genes were also analyzed using a phylogenetic network. Analysis was run in SplitsTree4 (version 4.18.1) [90] using the Neighbor-net method [91] based on uncorrected p-distances. The Phi test [92] was conducted to test for an overall evidence of recombination events within and among MLST genes.

### Fluorescence in situ hybridization

*Wolbachia* localization within host tissues was studied using the probe W2 (5’-CTTCTGTGAGTACCGTCATTATC-3’) [71], targeting the *Wolbachia* 16S rRNA, labelled on the 5’ end with Cy3. The probe W2 was used in combination with the universal bacterial probe EUB338 (5’-GCTGCCTCCCGTAGGAGT-3’) or the probe CFB319 (5’-TGGTCCGTGTCTCAGTAC-3’) targeting the primary symbiont *Sulcia muelleri* 16S rRNA [72]. The last two probes were labelled on the 5’ end with FAM. Cell nuclei were counterstained with DAPI. Live *P*. *spumarius* adult females were numb at -20°C and dissected at room temperature in ethanol 70% at a stereomicroscope (40X). The head was separated from the rest of the body and then dissected to extract the salivary glands. Ovaries and bacteriomes were extracted from the abdomen. Ovaries, bacteriomes and salivary glands were separately subjected to the whole-mount FISH method by [93] with modifications. Samples were fixed in Carnoy’s solution for 24 h, decolorized in 6% H_2_O_2_ in ethanol for 24–48 h, washed in absolute ethanol, and hybridized for 24 h at 25°C. Hybridization buffer (20 mM Tris-HCl [pH 8.0], 0.9 M NaCl, 0.01% SDS, 30% formamide) contained 10 pmol/ml of each fluorescent probe. Then, stained samples were rinsed in washing buffer (SSC 20X, 0.01% SDS) and mounted on slides in Vectashield antifade mounting medium with DAPI (1.5 μg/ml) (Vector Laboratories, Burlingame, CA, USA). Slides were observed and photographed at a Leica DM6000 B fluorescence microscope (Leica Biosystems, Nussloch, Germany) and images were processed with the Leica Application Suite X (LAS X). The specificity of the observed signals was verified by comparing the probe-treated individuals infected by *Wolbachia* (sampled from the infected populations of the Alto Adige region) with negative controls made by probe-treated uninfected individuals (sampled from the *Wolbachia*-free populations of the Campania region) and no-probe controls. The occurrence or absence of *Wolbachia* infection in each individual used for FISH experiments was confirmed by running PCR with *Wolbachia* specific primers as described above on DNA extracted from the rest of body parts not used for FISH slide preparation.

## Results

### *Philaenus spumarius* molecular typing and phylogeographic structure

The *COI* gene sequencing of 265 individuals of *P*. *spumarius* showed the occurrence of 75 haplotypes across Italy including 13 already reported by [46] (S2 Table). The median-joining haplotype network developed by combining the Italian haplotypes with the global haplotypes by [46] confirmed the division of *P*. *spumarius* into three main mitochondrial groups, namely the eastern-Mediterranean, western-Mediterranean and NE lineages, each with a specific geographic distribution (S1 Fig). Most haplotypes of the eastern-Mediterranean lineage showed a pattern of geographic isolation. The most frequent haplotype H49 and the majority of its derived haplotypes were recorded only from Italy. The second most common haplotype H57 and most of its derived haplotypes came predominantly from the Balkans, although some of them are also present elsewhere, including Italy. Haplotypes of the western-Mediterranean lineage showed a wider geographical distribution and some of them were shared among different geographic areas. For example, H29, the dominant haplotype in the Iberian Peninsula, is also present in Italy, Western Europe and Morocco. However, H32, the dominant western-Mediterranean haplotype in Italy, does not occur elsewhere. Haplotypes of the NE lineage were distributed over a wide geographic area, with the exception of southern Europe, as this lineage is present up to the alpine mountain range of northern Italy (Alto Adige region in this study). It was not possible to attribute unequivocally the haplotypes H73 and H107 (two individuals each) to either the eastern-Mediterranean or the western-Mediterranean lineage on the basis of the haplotype network.

**Fig 2:**
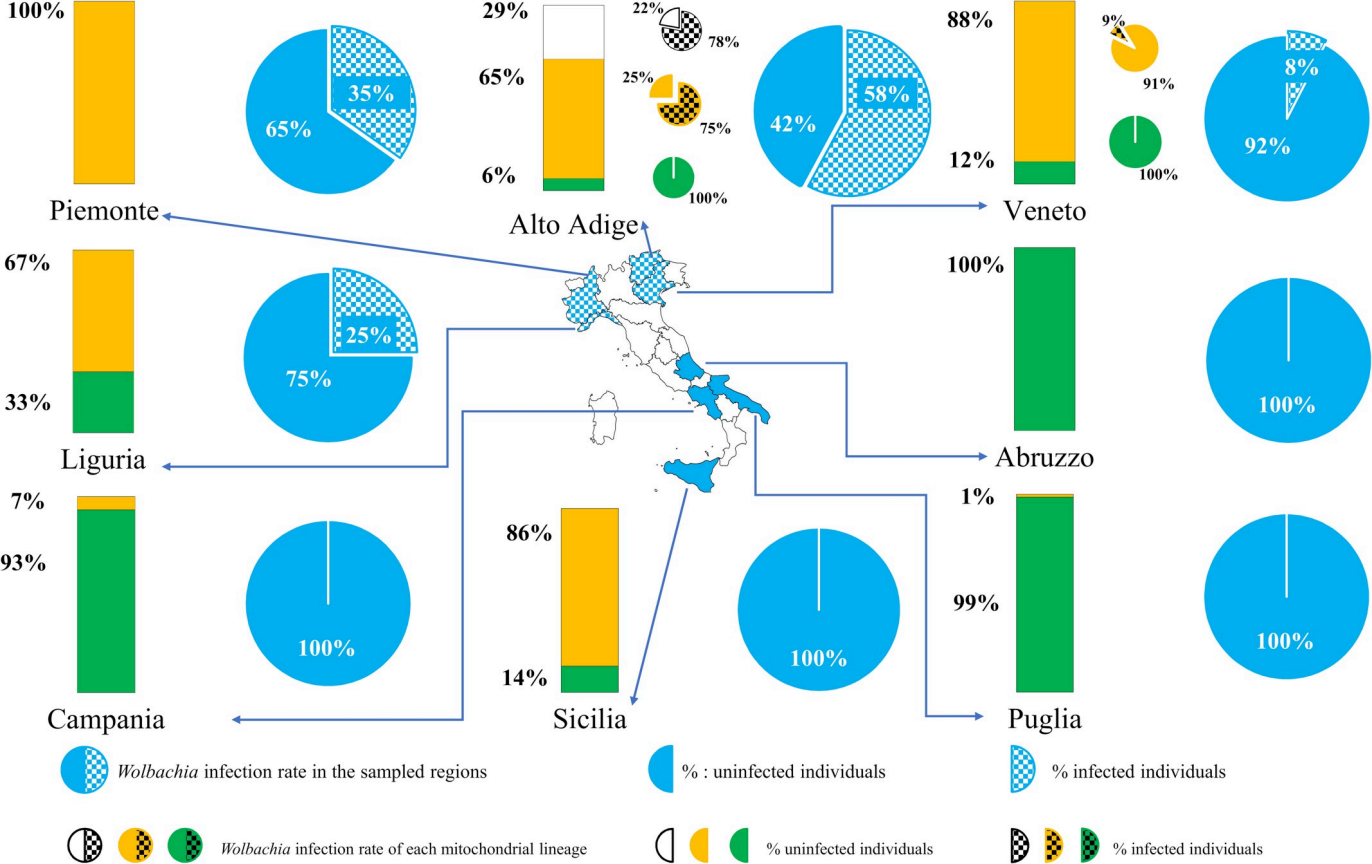
Frequency of mitochondrial lineages of *Philaenus spumarius* (histograms) and *Wolbachia* infection rate (cakes) in the sampled regions of Italy. The percentage of infected individuals is the slice of cake colored with checkered texture. Similarly, regions with populations of *P*. *spumarius* infected by *Wolbachia* are colored with checkered texture on the map. The blue big cakes represent the *Wolbachia* infection rate calculated on the total number of individuals sampled in each region. The small cakes represent the *Wolbachia* infection rate calculated on the individuals of each mitochondrial lineage. The map of Italy by Sinigagl (https://commons.wikimedia.org/wiki/File:Italy_template_blank.png) is licensed under CC BY-SA 3.0; it is similar but not identical (it differs for the colored regions) to the original map and is therefore for illustrative purposes only.

Each of the three lineages showed a specific geographic distribution within Italy (Figs 1A and 2, S2 Fig, S2 and S11 Tables). Individuals of the eastern-Mediterranean lineage were prevalent in southern and central Italy (87% and 100%, respectively), while those of the western-Mediterranean lineages represented 85% of individuals collected in northern Italy. The Sicilia region was an exception in southern Italy, with 80% of individuals belonging to the western-Mediterranean lineage. Among the 75 haplotypes recorded from Italy some were largely prevalent and many others derived from them, differing by only 1–3 mutations (Fig 1A). Most haplotypes were restricted to a specific geographic region, while 12 were shared by different regions. Fifty-six haplotypes were recorded only once. The dominant haplotypes were H32 and H49, each one accounting for 21.9% of total individuals.

Within the eastern-Mediterranean lineage, 45 different haplotypes were identified out of 127 individuals. The haplotype H49 accounted for 48.8% of eastern-Mediterranean individuals and showed the widest distribution across Italy. H49 was found in eight regions, from the southernmost (Sicilia) to the northernmost (Alto Adige) parts of Italy. The other haplotypes of the eastern-Mediterranean lineage were all represented by 1–2 individuals, except for H53 which reached 5.3% and 11.8% of total and eastern-Mediterranean individuals, respectively. This haplotype was recorded in five regions, from south (including Sicilia) to north Italy.

The number of haplotypes identified within the western-Mediterranean lineage was lower (21 haplotypes in 142 individuals) than in the eastern-Mediterranean lineage, and three haplotypes (H29, H32 and H34) accounted for 77% of total individuals. Haplotype H32 was found in all sampled regions of northern Italy, representing 43.6% of the western-Mediterranean individuals. The haplotype H29 was recorded in six regions, from north to south Italy (including Sicilia) and accounted for 8.8% and 17.6% of total and western-Mediterranean individuals, respectively. The haplotype H34 was recorded only in two regions of northern Italy and accounted for 8.2% and 16.2% of total and western-Mediterranean individuals, respectively.

The haplotypes of the NE lineage were the least represented in Italy. They were not found in south-central Italy and represented only 6% of the haplotypes in northern Italy. In particular, they were found exclusively in the northernmost part of Italy (Alps of Alto Adige region) where they accounted for 29% of total individuals. In this area the western-Mediterranean haplotypes were the dominant ones (64.5%) with the most common H24 and H95 restricted to this specific region.

Most populations of *P*. *spumarius* were characterized by the occurrence of more than one haplotype and admixtures of different lineages were found throughout northern Italy (S2 Table). Conversely, in south-central Italy almost all the populations were represented exclusively by haplotypes of the eastern-Mediterranean lineage. The exceptions were the populations Ca1 and Pu2, with a few western-Mediterranean individuals, and Si3, in which the western-Mediterranean individuals were prevalent (S2 Table). The highest number of haplotypes (eight) was scored for the *Wolbachia*-infected Aa2 population, an admixture of haplotypes of the three mitochondrial lineages.

### Prevalence of *Wolbachia* in Italian populations of *Philaenus spumarius*

A strong association between the infection status and the geographic distribution of host populations was observed. All 37 populations of *P*. *spumarius* sampled in south-central Italy (a total of 521 individuals) resulted uninfected. In northern Italy, *Wolbachia* was found in 11 out of 17 populations (64.7%) with an average infection rate of 40.5% (calculated on 262 individuals analyzed) (Table 1 and S2 Fig). The infection rate was variable among the populations of northern Italy (Table 1, Fig 2) and the differences among northern regions were significant (G-test of independence, G = 56.75, df = 3, P<0.001). The highest infection rates were recorded in populations sampled at the highest altitudes (on the Alps) in the northernmost region (Alto Adige). In this area, the infection rate was 27% at 1000 m, 80% at 1200 m and 76% at 1848 m asl (G-test of independence, G = 37.85, df = 2, P<0.001). In the remaining regions, *P*. *spumarius* populations, sampled between 35 and 379 m asl, showed infection rates ranging between 0 and 53%, without any relationship to altitude. There were no significant differences in the infection rates between sexes (Table 1) when data from all infected populations sampled in northern Italy were aggregated, with 45% and 33% of infected females and male, respectively (G-test of independence, G = 3.61, df = 1, P = 0.06). Likewise, even limiting the comparison to populations in individual regions, the infection rate did not differ significantly between females and males. Finally, diagnostic PCR revealed that half of the infected individuals sampled in Alto Adige and Piemonte had *Wolbachia* localized in the head (Table 2) and more frequently in females than in males (G-test of independence, G = 7,01, d.f. = 1, P = 0.008).

### Sex ratio in the populations of *Philaenus spumarius*

The sex ratio of *P*. *spumarius* (S3 Table) did not differ significantly among the *Wolbachia*-infected populations of northern Italy. The proportion of females ranged between 52.8% and 73.3% and was not related to the infection rate. The sex ratio was highly variable among the uninfected populations of southern Italy. The proportion of females ranged between 33% and 88% and there were significant difference among the populations of the Puglia region and when all populations of southern Italy were compared. Analysis of aggregated data showed no significant differences in the proportion of females among the geographic regions, independently by the infection of *Wolbachia*. Overall, these data did not show substantial differences in the sex ratio of *Wolbachia*-infected and unifected populations of *P*. *spumarius*.

### Diversity of *Philaenus spumarius* mtDNA

The population genetic parameters of mtDNA revealed a high diversity within the geographic groups of *P*. *spumarius*, without consistent differences between northern and south-central Italy, when the populations were grouped by either region of origin or by geographic macro-area (S4 Table). The comparison between geographic groups of populations did not show a direct relationship between the increase in the infection rate and the reduction in mitochondrial molecular diversity. The regional group including the populations with the highest *Wolbachia* infection rates (Alto Adige, Table 1) showed the highest values of molecular diversity parameters. This result was partly explained by the increase in genetic variability due to the distinctive presence in this region of haplotypes of the NE lineage in admixture with haplotypes of the other two lineages (Fig 2, S2 and S11 Tables). The NE lineage haplotypes, other than diverge genetically from the eastern-Mediterranean and western Mediterranean lineages, showed a higher within-group genetic distance (S12 Table). The populations of Piemonte and Veneto, unlike those of the Alto Adige, were more homogeneous genetically as they were almost exclusively characterized by haplotypes of the western-Mediterranean lineage (except for few haplotypes of the eastern-Mediterranean lineage within the populations of Veneto) (S2 and S11 Tables). Nevertheless, even when the NE haplotypes were excluded from the analysis and the regional groups were compared by considering only the haplotypes of the other two lineages, molecular diversity parameters of the Alto Adige group resulted always higher than those estimated for the other regional groups either infected or uninfected (S4 Table). Values of molecular diversity parameters were much lower for the populations of Piemonte and Veneto, characterized by *Wolbachia* infection rates considerably lower than those of the Alto Adige. The same variation in molecular diversity observed among the populations of the northern regions was found among the regions of south-central Italy where all populations were uninfected. The populations of Puglia were comparable to those of Alto Adige in terms of haplotype diversity (*Hd* = 0.889, the highest value in south-central Italy) but showed halved values of nucleotide diversity and mean number of pairwise differences, probably due to the almost exclusive presence of haplotypes of the eastern-Mediterranean lineage. The lowest molecular diversity was found in Sicilia (S4 Table). In this region, the populations turned out to be unique among those of southern Italy for being almost exclusively represented by western-Mediterranean haplotypes (11 of 13 individuals) and genetically uniform (10 individuals were H29 haplotype) (S2 and S5 Tables).

Neutrality tests, performed on the regional groups of uninfected *P*. *spumarius* populations of south-central Italy, produced significant negative statistics, suggestive of population expansion (S4 Table). The neutrality test statistics of the *Wolbachia-*infected regional groups of *P*. *spumarius* from northern Italy did not deviate significantly from neutral expectation, except for the negative estimates of Fu’s FS for the Piemonte and Veneto population groups (S4 Table). Thus, no evidence of selective sweep emerged for the group of highly infected populations of Alto Adige, while partial evidence seemed to exist for the populations of Piemonte and Veneto. However, within the Piemonte group, molecular diversity parameters did not substantially differentiate between infected and uninfected haplotypes (S6 Table). Further, for the populations of Piemonte and Veneto, the negative estimates of Fu’s FS were statistically significant only for the uninfected haplotypes. On a larger geographic scale (Italy and northern Italy), no substantial difference in molecular diversity parameters emerged between infected and uninfected haplotypes or molecular diversity was greater for infected than for uninfected haplotypes. Finally, the neutrality tests statistics showed a significant negative deviation from the equilibrium for the uninfected but not the infected haplotypes (S6 Table).

When the overall set of Italian haplotypes was grouped by mitochondrial lineage, the highest molecular diversity was found in the NE lineage, while the diversity parameters did not differ between the eastern-Mediterranean and the western-Mediterranean lineage (S5 Table). Tajima’s D and Fu’s FS statistics showed significant negative deviation from expected equilibrium for the eastern-Mediterranean and western-Mediterranean lineages but not for the NE lineage despite the latter showing the highest rate of infection (Table 4 and S2 Fig). An identical result was obtained by running the analysis only on the haplotypes of northern Italy. When the analysis was focused on the regional groups (S5 Table), the molecular diversity parameters of the western-Mediterranean individuals were higher for Alto Adige, the group with the highest infection rate (Table 4 and Fig 2). Further, focusing the analysis on the infected and uninfected haplotypes of the western-Mediterranean lineage, neutrality tests were statistically significant only for the uninfected haplotypes (S6 Table).

**Table 4:** *Wolbachia* infection of mitochondrial lineages of *Philaenus spumarius*.

Geographic area	Mitochondrial lineage	n	*Wolbachia* infection rate
**Italy**	eastern-Mediterranean	78	1.28%
	western-Mediterranean	126	26.98%
	north-eastern	9	77.78%
	total	213	19.72%
	south-western^**†**^	204	17.16%
**Northern Italy**	eastern-Mediterranean	10	10%
	western-Mediterranean	114	29.82%
	north-eastern	9	77.78%
	total	133	31.58%
	south-western^**†**^	124	28.23%
**Alto Adige**	eastern-Mediterranean	2	0%
	western-Mediterranean	20	75%
	north-eastern	9	77.78%
	total	31	70.97%
	south-western^**†**^	22	68.18%
**Piemonte**	eastern-Mediterranean	0	-
	western-Mediterranean	38	36.84%
**Veneto**	eastern-Mediterranean	7	0%
	western-Mediterranean	55	9.09%
	total	62	8.06%

^†^The south-western lineage includes the eastern-Mediterranean and the western-Mediterranean lineages.

The AMOVA (S7 Table) performed for the populations of *P*. *spumarius* grouped by region of origin showed that more than 57% of the genetic variation is significantly explained by genetic differences within populations while the geographic grouping accounted for only 39% of the genetic variation (P<0.001). Similarly, a weak geographic structure emerged when the AMOVA was run by grouping the populations by macro-area of origin (south-central Italy versus northern Italy). When the AMOVA was run by grouping the populations according to the presence or absence of *Wolbachia* infection, only a small percentage of genetic variation was explained by the infection status (P<0.001) and 54% of the variation was explained by genetic differences within populations. Similarly, for each geographic area (S8 Table) or considering only the western-Mediterranean lineage (S9 Table), the AMOVA run by grouping the haplotypes by presence or absence of *Wolbachia* has always shown a very small component of the genetic variation associated with the infection status. The only exception were the individuals of the western-Mediterranean lineage of the Alto Adige populations, with 43% of the genetic variation explained by the infection status. Overall, AMOVA supports a weak impact of *Wolbachia* infection on population genetic variability.

### *Wolbachia* molecular typing and phylogenetic analysis

**Fig 3:**
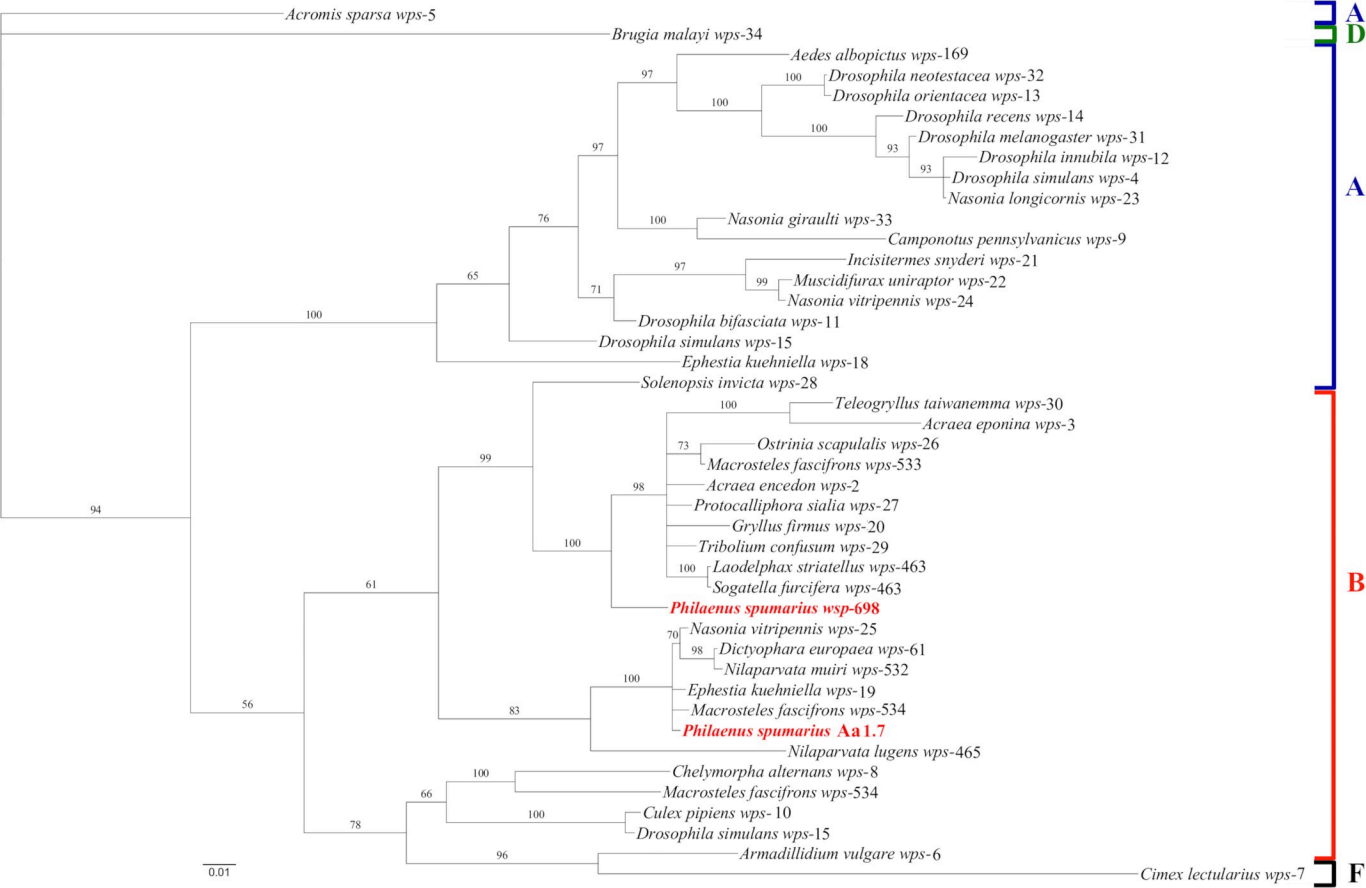
Bayesian phylogenetic tree for *wsp* gene sequences of *Wolbachia*. Strains of *Wolbachia* found in *Philaenus spumarius* and sequenced in this work are shown in bold red. Reference *Wolbachia* strains were imported from the MLST database. *Wolbachia* strains are indicated with the name of the host species followed by the number of the *wsp* allelic variant. Based on the information reported in the MLST database for the reference strains, the letters on the right indicates the *Wolbachia* supergroups.

Based on the nucleotide sequence of *wsp* gene and the amino acid sequence of the hypervariable regions of the WSP protein, two allelic variants were found in the Italian individuals of *P*. *spumarius*. The allelic variant *wsp*-698 occurred in all but one spittlebug (Table 3 and S10 Table) and was associated to two butterfly species in the MLST database (namely, *Carterocephalus palaemon* -Pallas-, id 479, ST-353 and *Hyponephele lycaon* Küns, id 486, ST-359). We were not able to sequence the HVR1 region of the other *wsp* allelic variant, so it was not formally included in the *Wolbachia* MLST database. Based on the *wsp* gene phylogeny (Fig 3), *Wolbachia* symbionts of *P*. *spumarius* grouped in two different highly supported clades comprising reference sequences of supergroup B.

**Fig 4:**
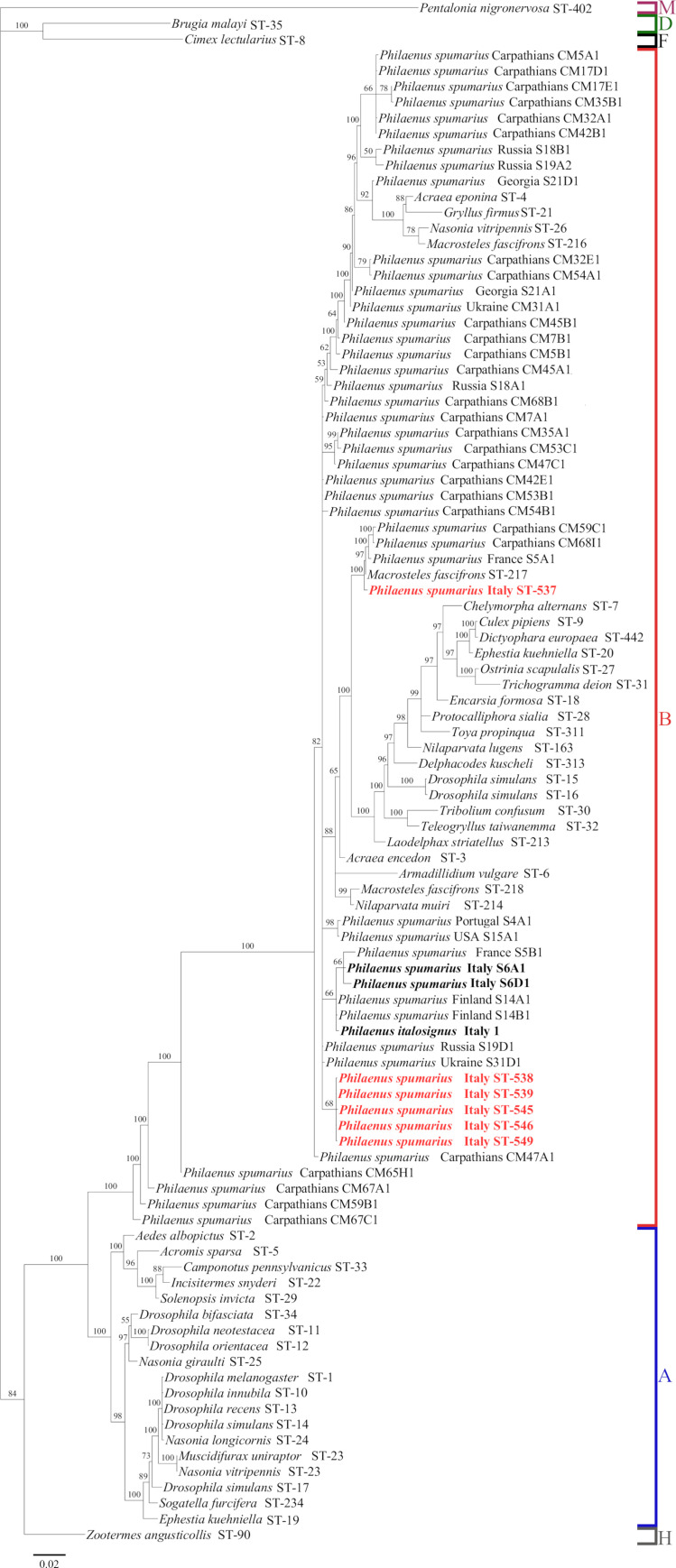
Bayesian phylogenetic tree for concatenated MLST gene sequences of *Wolbachia*. *Wolbachia* STs found in *Philaenus spumarius* and sequenced in this work are shown in bold red. Reference *Wolbachia* STs were imported from the MLST database and are indicated on the tree with the name of the host species and the number of the ST. *Wolbachia* strains of *P*. *spumarius* by [53] are reported with the original strain number (ST number not available in the MLST database) and those from Italy are in bold. Based on the information reported in the MLST database for the reference STs, the letters on the right indicates the *Wolbachia* supergroups.

MLST typing revealed much more diversity than the *wsp* gene. *Wolbachia* infecting our Italian samples of *P*. *spumarius* were classified into six new STs in the MLST database (Table 3 and S10 Table). The highest allelic variation was found in the *coxA* and *hcpA* genes with four and three variants, respectively. Two new allelic variants (301 and 302) were found for *coxA* gene, and one (470) for *fbpA* gene. The five *Wolbachia* STs with identical *wsp* allelic variant (STs-538, 539, 545, 546 and 549) also showed invariant *gatB*, *ftsZ* and *fbpA* genes. Only single infections of *Wolbachia* were identified (no double peaks were detected in gene sequence chromatograms). BI analysis of concatenated MLST gene sequences grouped these STs in a single clade whose phylogenetic relationship was unresolved (Fig 4). The high genetic relatedness between Italian STs in this clade emerged also by the very low pairwise genetic divergence between the concatenated gene sequences (uncorrected p-distance ranging from 0.048 to 0.29%) (S13 Table). *Wolbachia* ST-537 grouped in a different, highly supported, clade (mirroring the result of the *wsp* gene tree), including *Wolbachia* strains of *P*. *spumarius* from Carpathians and France, and the symbiont of a leafhopper species. *Wolbachia* ST-537 diverged by 2.2–2.5% (uncorrected p-distance) from the other *Wolbachia* STs found in Italian *P*. *spumarius* (S13 Table), sharing with most of them only the allele 159 of the *hcpA* gene. ST-537 showed a low genetic divergence with the strains from Carpathians and France (uncorrected p-distance, 0.46–0.61%). Finally, the Italian STs from this study diverged from the *Wolbachia* strains (S6A1 and S6D1 in Fig 4) of Italian *P*. *spumarius* collected in the region Friuli-Venezia-Giulia by [53] (uncorrected p-distance, 1.8–2.2% and 0.8–1.5% for pairwise comparisons with ST 537 and the other STs, respectively). BI analysis of concatenated MLST genes (Fig 4) showed that all *Wolbachia* STs of Italian *P*. *spumarius* identified in this study clustered in the supergroup B. Congruent results were obtained from the single gene phylogenies (S3 Fig). The exception was *coxA* gene, for which the monophily of supergroup B was not supported by BI analysis. However, *coxA* allelic variants of Italian *P*. *spumarius* were closely related to allelic variants of reference STs of supergroup B. Results similar to those of the BI analysis were obtained with the ML analysis of concatenated and single MLST genes (S4 and S5 Figs).

**Fig 5:**
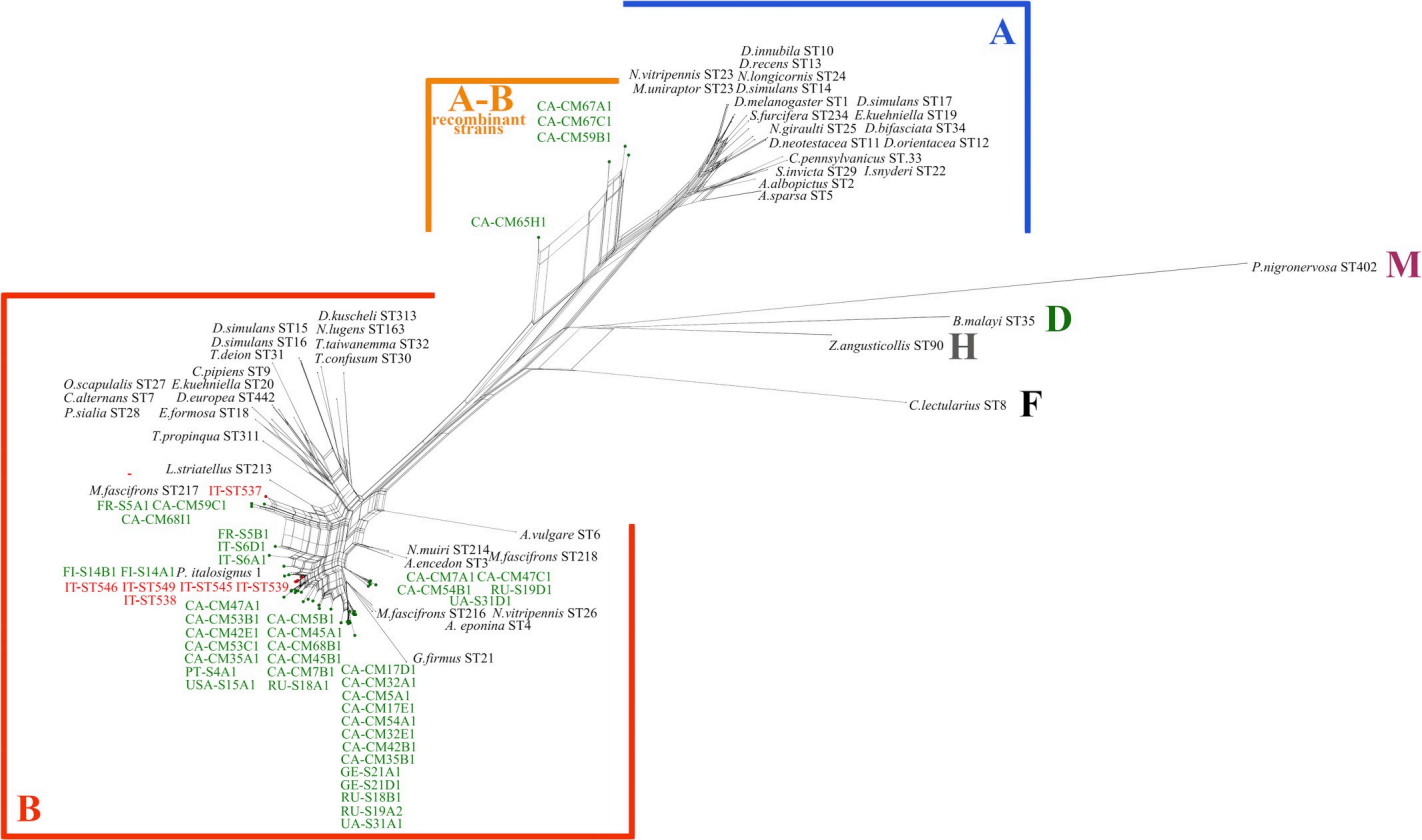
Neighbour-net phylogenetic network based on concatenated MLST gene sequences of *Wolbachia*. *Wolbachia* STs found in *Philaenus spumarius* and sequenced in this work are shown in red (IT means Italy). *Wolbachia* strains of *P*. *spumarius* by [53] are shown in green with the original strain number preceded by the acronym of the Country of origin. Reference *Wolbachia* STs were imported from the MLST database and are indicated on the tree with the name of the host species and the number of the ST. The capital letters indicates the *Wolbachia* supergroups.

Considering the global *Wolbachia* strains of *P*. *spumarius* [53] included in the analysis, the BI tree of concatenated MLST genes showed the occurrence of different lineages, most of them clearly embedded in the supergroup B. In addition, five *Wolbachia* strains from Carpathians (CM47A1, CM59B1, CM65H1, CM67A1 and CM67C1) [43] had a sister relationship with the supergroup B (Fig 4), forming with it a highly supported clade. For these strains, BI phylogenetic analysis revealed some incongruence in terms of supergroup assignment between concatenated and single MLST genes. Indeed, single gene phylogeny showed that few alleles of *gatB*, *coxA* and *hcpA* genes grouped in or have a sister relationship with the *Wolbachia* supergroup A (S3 Fig). As for *fbpA* gene, most alleles were classified in the supergroup B, and a few alleles were not assigned to any of the two supergroups (S3 Fig). The phylogeny of concatenated genes produced by the ML analysis (S4 Fig) supported a sister relationship with the supergroup A of four *Wolbachia* strains from Carpathian basal to supergroup B in the BI analysis and an analogous result was produced by the ML phylogenies of the single *gatB*, *coxA* and *hcpA* genes (S5 Fig). Both *ftsZ* phylogenies produced with BI or ML analysis significantly placed all *Wolbachia* symbionts of *P*. *spumarius* into supergroup B. The topological incongruence between concatenated and single MSLT gene trees, as well as between BI and ML trees based on concatenated genes, were the consequence of recombination events. Indeed, the network analysis performed on concatenated MLST genes allowed to visualize, through the occurrence of multiple boxes, probable recombination events (Fig 5 and S6 Fig) and the phi test did find statistically significant evidence for recombination (P = 0) for both the whole and *P*. *spumarius* data set. Despite recombination, the result of the network analysis (Fig 5) was congruent with that of the BI and ML analyses. Indeed, the *Wolbachia* strains of *P*. *spumarius* were grouped in the supergroup B with the exception of four strains from Carpathians. The latter were recombinant strains, characterized by the *gatB*, *coxA*, *hcpA* and *fbpA* alleles of supergroup A and the *ftsZ* allele of supergroup B (S7 Fig). These recombinant strains formed an intermediate group in the network, closer to supergroup A (Fig 5), mirroring the result of the ML tree (S4 Fig).

### Association of *Wolbachia* with mitochondrial haplotypes of *Philaenus spumarius*

The infection rate differed significantly between the three mitochondrial lineages, when comparisons referred both to Italy (Fisher’s exact test of independence, P = 2.3E-10) or northern Italy, the sole geographic area with *P*. *spumarius* populations infected by *Wolbachia* (Fisher’s exact test of independence, P = 0.004) (Table 4 and S2 Fig). However, in northern Italy, there was no significant difference between the infection rates of the eastern-Mediterranean and western-Mediterranean lineage (10% and 29.8%, respectively) (Fisher’s exact test of independence, P = 0.28). The infection rate of the NE lineages (77.8%) was the highest. When the comparison was focused on the individuals sampled exclusively from Alto Adige, the region with the highest *Wolbachia*-infection rates (Table 4 and Fig 2), no significant difference emerged between the NE lineage and the western-Mediterranean lineage (Fisher’s exact test of independence, P = 1) or the whole SW lineage (Fisher’s exact test of independence, P = 0.69).

Focusing only on individuals of the western-Mediterranean lineage, the *Wolbachia* infection rate differed significantly among Veneto, Piemonte and Alto Adige (9.1%, 36.8% and 75%, respectively) (Fisher’s exact test, P = 1.3E-07), with higher values as longitude (Veneto and Piemonte vs Alto Adige) and altitude (Veneto, 35–115 m asl; Piemonte, 185–379 m asl; AltoAdige, 1000–1848 m asl) increased (Table 4). Individuals of the western-Mediterranean lineage sampled in southern Italy were uninfected (Fig 2, S2 Fig and S2 Table). The dominant haplotypes of the western-Mediterranean lineage were characterized by variability in the infection status (Fig 1B) both within and between populations (S2 Table). *Wolbachia*-infected and uninfected individuals of the H24 haplotype were found within the Aa1 and Aa3 populations, and those of the H32 haplotype within the Pi1, Pi6, Ve4 and Ve5 populations. The haplotype H32 was always uninfected in Pi3, Pi5, Li1, Ve1 and Aa2 populations. The haplotype H29 was infected or uninfected in Piemonte depending on the population and always uninfected in Veneto (Ve6) and in southern Italy (Ca1 and Si3). Overall, 72.7% of H29 individuals sampled in northern Italy (n = 11) were infected by *Wolbachia*, while those sampled in south-central Italy (n = 10) were not (Fisher’s exact test, P = 0.001).

The association between *Wolbachia* strain and *P*. *spumarius* haplotype was found to be non-specific. Three *Wolbachia* STs (ST-538, -539 and -549) were each associated with more than one haplotype, belonging to different mitochondrial lineages (Table 3). *Wolbachia* ST-539, found in populations from Alto Adige and Piemonte regions, was the prevalent strain (45% of individuals) and was associated with five haplotypes of the western-Mediterranean lineage and two haplotypes of the NE lineage. The haplotype H06 was polymorphic for the infection within the Aa1 population, being singly infected by *Wolbachia* ST-539 or ST-546. *Wolbachia* STs 537, 538, 545 and 546 were exclusively associated to haplotypes collected in Alto Adige while ST-549 was found in all sampled regions of northern Italy except Alto Adige.

### Fluorescence in situ hybridization

**Fig 6:**
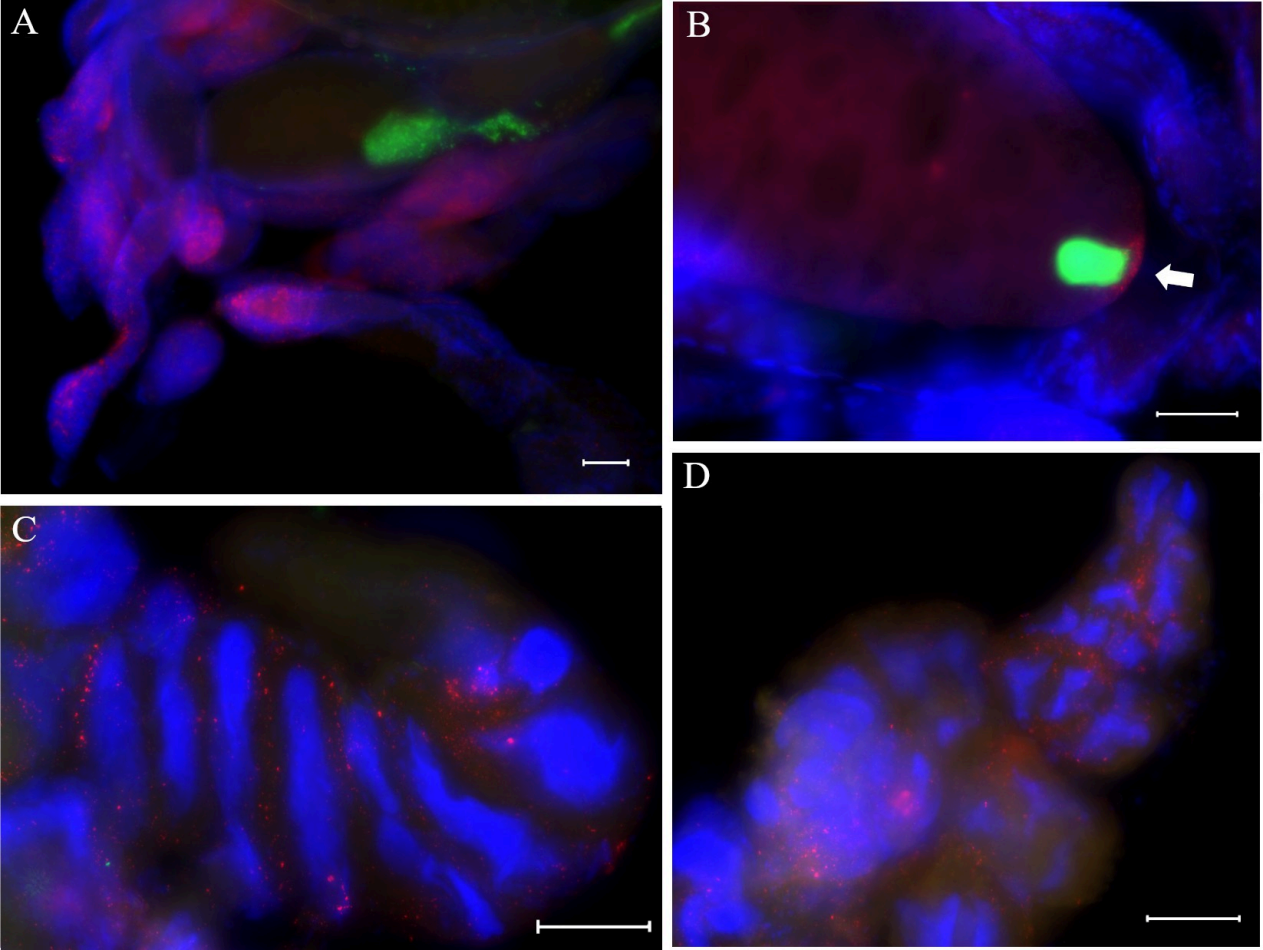
Fluorescence in-situ hybridization of *Philaenus spumarius*. Ovarioles (A and B) and salivary glands (C and D) stained by a *Wolbachia* and a *Sulcia* specific probes. (A) Distribution of *Wolbachia* bacteria (bright red) within telotrophic ovarioles and *Sulcia* primary symbionts (green) penetrated into developing oocytes. (B) *Wolbachia* symbionts (bright red, arrow) and the ball-shaped mass of *Sulcia* symbionts (green) localized at the posterior pole of a developed oocyte; autofluorescence of ooplasm is visible. (C and D) *Wolbachia* bacteria (bright red) distributed within an upper (C) and a ventral (D) salivary gland. DAPI-stained nuclei are blue. Bars, 100 μm.

*Wolbachia* bacteria were spread throughout the telotrophic ovarioles, with the highest concentration in the germarium (Fig 6A and S8 Fig). In the developing oocyte, *Wolbachia* bacteria were distributed in the ooplasm (S8 Fig) and concentrated at the posterior pole at late oogenesis (Fig 6B). *Wolbachia* showed a different localization from that of the primary symbionts. The latter were present only within the terminal oocytes where they penetrated through the posterior pole to form a ball-shaped mass (symbiont ball) (Fig 6A, 6B, S8 and S9 Figs). There was no evidence of *Wolbachia* in the bacteriomes, where the occurrence of primary symbionts was evident inside the bacteriocytes (S9 Fig). Finally, *Wolbachia* bacteria were found within the salivary glands of *Wolbachia*-infected adult females (Fig 6C and 6D). Negative controls (no-probe treated individuals and probe-treated *Wolbachia*-free individuals) did not display signals (S9 Fig), confirming the specificity of the signals detected in infected individuals.

## Discussion

*Wolbachia* infections in *P*. *spumarius* occur throughout the host range [53, 54] but the prevalence within host populations and the role the symbiont plays on host ecology remain largely unknown. Here, we investigated the prevalence of *Wolbachia* infection in relation to the geographic distribution and genetic diversity of *P*. *spumarius* in Italy. In this Country the insect raised to the status of key pest being the main vector of *X*. *fastidiosa* subspecies *pauca*, which is devastating the olive growing [39, 40]. The results highlight some factors influencing the ecology of *Wolbachia* within the Italian populations of *P*. *spumarius*, the knowledge of which is of interest for a possible use of *Wolbachia* to control the spread of *X*. *fastidiosa*.

Across Italy, *Wolbachia* infection in *P*. *spumarius* populations followed a geographic pattern closely associated with the geographic distribution of the three mitochondrial lineages. *Wolbachia* was detected exclusively within the populations of northern Italy, where the western-Mediterranean lineage was dominant (85% of individuals). Conversely, all populations sampled in south-central Italy were uninfected. In that area, the individuals of the eastern-Mediterranean lineage were prevalent (87%). The exception was Sicilia, the southernmost region, characterized by the dominance of the western-Mediterranean lineage (86%) and by a lower molecular diversity of *P*. *spumarius* compared to other regions of Italy. An exception that is explained by the founder effect and geographic isolation, being Sicilia an island. The prevalence of *Wolbachia* infection varied between the geographic regions of northern Italy and between *P*. *spumarius* populations in each region. The highest rates were recorded on Alps in Alto Adige, the northernmost and coldest region, where the populations differed from others in northern Italy due to the specific occurrence (29%) of individuals of the NE lineage (absent elsewhere). We observed a tendency for the infection to increase with the altitude, with the highest rates found at 1200 m (80%) and 1800 m asl (76%). Our results are in line with those by [53]. These authors, analyzing only a few individuals, did not find *Wolbachia* infection in the populations (SW lineage) of south-central Italy while in north-eastern Italy, on Alps of Friuli-Venezia-Giulia region, two individuals of the NE lineage were infected and two of the SW lineage were uninfected.

Overall, the genetic diversity of the Italian populations of *P*. *spumarius*, estimated on the mtDNA, was high and mostly explained by genetic differences within populations rather than by geographic groupings of populations. All populations were admixtures of multiple haplotypes, often unrelated and belonging to two (three in a single case) mitochondrial lineages. Of the 75 haplotypes found in Italy, 12 were shared among populations of different regions, from north to south. For example, the dominant haplotype H49 (eastern-Mediterranean lineage) was distributed in all regions of south-central Italy and two northern regions, from sea level to 1400 m asl. The distribution and frequency of some haplotypes (e.g. H49, closely related to Balkans haplotypes, and H29, the most common in the Iberian Peninsula) support the hypothesis [45, 46] that Italian populations would have originated with the expansion of the eastern-Mediterranean lineage from Balkans and the western-Mediterranean lineages from Iberian Peninsula to northern Italy. The Alps of the northeastern regions of Italy (Alto Adige in this study, Friuli-Venezia-Giulia in [45]) are a contact zone between the NE and the SW lineage, which would act as a barrier to the introduction of the NE lineage after its expansion from the Anatolia/Caucasus/western Asia [45].

What could be the factors influencing the ecology of *Wolbachia* in the Italian populations of *P*. *spumarius*? The spread of vertically transmitted symbionts and the prevalence of their infections within host populations depend on several factors, including the efficiency of maternal transmission, the phenotypic effect on the host and the relative fitness of infected hosts [4, 94], which in turn can be affected by the interaction between symbiont genotype, host genotype and environment [95–101]. Among the environmental factors, temperature plays a fundamental role in determining the persistence of *Wolbachia* within host populations and the evolution of the symbiotic interaction. The type of response can vary between *Wolbachia* strains, which may be susceptible to heat in some host species and cold in others [99, 101, 102]. Consequently, the infection rate and the phenotypic effect of *Wolbachia* may vary in the distribution range of a host species and the differences observed between geographic areas may be related to climatic parameters and/or the genetic differentiation of the host populations. Our results would lead to minimize the influence of *P*. *spumarius* genetic background on *Wolbachia* spread. Indeed, if it is true that on the total of the haplotypes analyzed the infection rate differed among the three mitochondrial lineages, the difference between eastern-Mediterranean and western Mediterranean lineage was no longer significant when the analysis was restricted to northern Italy. This result, however, cannot be conclusive, due to the small number of haplotypes of the eastern-Mediterranean lineage sampled in northern Italy. Finally, even focusing the analysis on Alto Adige, the region with the highest prevalence of *Wolbachia*, the impact of the mitochondrial lineage on the infection rate was negligible, with no significant difference between western-Mediterranean and NE lineage. In a previous study [53], the average infection rate was significantly different between the NE and the SW lineages (70% and 20%, respectively) over the entire geographic range of *P*. *spumarius*, with values comparable to those observed here for Italy. This difference emerged also in the Carpathians contact zone, where the infection rate was 93% for the NE lineage and 46% for the SW lineage. This is in contrast to the situation we found in the contact zone of Alto Adige, which could be explained by the genetic composition of the SW lineage, almost entirely represented by western-Mediterranean haplotypes (75% infection rate) and few uninfected eastern-Mediterranean haplotypes. The study by [53] did not distinguish the *Wolbachia* infection rate between the western-Mediterranean and the eastern-Mediterranean lineages, but graphically showed that in the Carpathians, all haplotypes of the western-Mediterranean and the NE lineages were infected, whilst those of the eastern-Mediterranean lineage had a low infection rate. Coherently, a recent study [54] showed absence of *Wolbachia* or low infection rates (4–15%) in populations of the eastern-Mediterranean lineage sampled from Greece, which were infected by multiple facultative endosymbionts with a prevalence of *Rickettsia*. This open the question to whether, in *P*. *spumarius*, competitive interactions between symbionts [103–105] could evolve to hinder a stable *Wolbachia* infection, which could be costly to specific host genotypes. Taken together, our Italy-wide data and those of [53, 54] might suggest that the eastern-Mediterranean lineage is actually less susceptible to *Wolbachia* infection. However, mitochondrial genes alone may not be fully informative to assess the interaction between *Wolbachia* and host genetic background. Although three analogous main evolutionary branches of *P*. *spumarius* emerged from the phylogeographic analysis of both mitochondrial and nuclear genes, a partial discrepancy was found between the two phylogeographic structures [45, 47, 49]. Indeed, some individuals with mitochondrial haplotype of the eastern-Mediterranean or of the western-Mediterranean lineage, clustered in the NE lineage of nuclear phylogeny. This may be the consequence of incomplete lineage sorting or of introgressive hybridization along contact zones [46, 48], a process that could also be driven by *Wolbachia* infections [106, 107]. The nuclear gene EF1α, which has been used in previous studies, appears not perfectly useful for large screening due to the low percentage of individuals that can be successfully sequenced [45, 49]. Additional nuclear genes or genomic data [48] will need to be considered in future studies to understand the relationship between *Wolbachia* infection and genetic diversity of *P*. *spumarius*.

Our results suggest that among environmental factors, prolonged periods of heat and drought, such as those characterizing the Mediterranean climate of south-central Italy, could play a key role in shaping the geographic distribution of *Wolbachia* among the Italian populations of *P*. *spumarius*. High temperatures, by pulling down the density of symbionts in the host, can negatively affect the efficiency of vertical transmission and consequently the prevalence of *Wolbachia* infections [94, 99, 102]. We got more than one piece of evidence supporting the impact of temperature. First, *P*. *spumarius* populations were always uninfected in south-central Italy, characterized by a Mediterranean climate with a hot summer season. Second, in northern Italy, the infection rate increased with increasing longitude and altitude, i.e. passing from temperate lowland and hilly areas to cold mountain areas. Finally, the haplotype H29 of the western-Mediterranean lineage, although highly infected in northern Italy, resulted always without *Wolbachia* in southern Italy, in three different regions. By setting up rearings of *Wolbachia*-infected *P*. *spumarius* from high elevation alpine sites under warm and dry conditions of Mediterranean areas would help in identifying the main driver of this symbiosis. It is worth noting that in cold environments ovarian parapause of *P*. *spumarius* females is very short or virtually absent and eggs are formed soon after female emergence. On the contrary, in Mediterranean areas, females undergo a long ovarial summer parapause [108, 109], lasting at least four months, before maturing eggs. It is conceivable that during this prolonged warm period, *Wolbachia*, if present, are lost and therefore vertical transmission via the egg is inefficient.

The diversity of *Wolbachia* in Italian populations of *P*. *spumarius* was characterized using sequence data and phylogenetic analyses of the *wsp* gene and the concatenated MLST genes [70]. The comparison with the sequences deposited in the MLST database indicated six new *Wolbachia* sequence types from *P*. *spumarius*. Five of them (STs 538, 539, 545, 546 and 549) were closely related based on concatenated MLST gene phylogeny and shared the same allelic variant of the *wsp* gene. *Wolbachia* ST-537, which was found in a single specimen of the NE lineage, had a different variant of the *wsp* gene and within the MSLT tree, grouped in a distantly related clade, including the *Wolbachia* strains of *P*. *spumarius* from Carpathians and France, and the symbiont of the leafhopper *Macrosteles fascifrons* (Stål). All six STs were classified in the *Wolbachia* supergroup B. Contrary to what reported from other geographic areas [53], we did not find neither double infections nor strains of the supergroup A. However, based on our phylogenetic analyses, the *Wolbachia* strains of *P*. *spumarius* from Carpathians, attributed by [43] to supergroup A, could not be unequivocally classifiable to one of the two supergroups. While BI analysis of concatenated MLST genes revealed a close relationship between these Carpathian strains and the supergroup B, this result was not congruent with the single *hcpA*, *gatB* and *coxA* gene phylogenies since few alleles grouped in or have sister relationship to supergroup A. The ML phylogeny of concatenated MLST genes was also incongruent with the result of BI analysis, because four of the five Carpathian strains, closely related to supergroup B in BI phylogeny, now showed a sister relationship to supergroup A. The conflicting signals produced by different phylogenies can reflect the presence of recombination events in MLST genes, which can occur between *Wolbachia* strains of the same supergroup or of different supergroups [84–88]. In fact, the network analysis confirmed a significant presence of recombination events between the concatenated MLST genes. Interestingly, in the network, the *Wolbachia* strains of *P*. *spumarius* split into three groups, including the A and the B supergroups, and an intermediate group consisting of A-B recombinant strains from Carpathians. The phylogenetic position of these strains will be better clarified through the analysis of other non-recombinant genes or a genomic approach [84, 110].

*Wolbachia* endosymbionts can influence the evolutionary dynamics of mitochondrial DNA of host populations. *Wolbachia* and mitochondria are simultaneously inherited through the egg cytoplasm, and if the infected individuals have a reproductive advantage over the uninfected ones, the spread of *Wolbachia* will sweep from the infected populations the mitochondrial haplotypes not associated with infection. The result will be the reduction in the mtDNA diversity, but not that of nuclear markers, of the infected populations and the increased frequency of the mitochondrial haplotype that benefits the most from the association with *Wolbachia* [76, 111]. Eventually, after a *Wolbachia* selective sweep, the mtDNA will evolve in a non-neutral way and a higher number of low frequency variants are expected in an infected population compared to uninfected one [77, 78, 119]. Contrary to expectations, we did not find a direct relationship between the reduction of the *COI* gene molecular diversity and the increasing of *Wolbachia* infection rate. Comparing the populations of the three analyzed regions of northern Italy, the highest value of genetic diversity was found for populations of Alto Adige, despite showing the highest infection rates. This finding and the absence of significant deviations from neutrality led us to ruling out that a recent selective sweep has occurred in the populations of northern Italy. In accordance, even when the haplotypes were grouped according to the mitochondrial lineage, molecular diversity parameters were not negatively related with the *Wolbachia* infection rate. Indeed, the highest molecular diversity (along with no deviation from neutral evolution) was estimated for the NE lineage, the group with the highest infection rate. Furthermore, either considering all haplotypes sampled in each geographic region or focusing the analysis only on the haplotypes of the western-Mediterranean lineage (the largest component of *P*. *spumarius* in northern Italy), infected individuals always showed greater molecular diversity than uninfected ones, and no deviation from neutrality. As for the populations of south-central Italy, mainly represented by eastern-Mediterranean haplotypes, the variation in molecular diversity among the four geographic groups of populations (uninfected) was similar to that found among the three geographic groups (infected by *Wolbachia*) of northern Italy. Consistently, molecular diversity parameters did not differ much between eastern-Mediterranean and western-Mediterranean lineages, despite they differed greatly in the rate of infection. In south-central Italy, neutrality tests showed significant deviations from the condition of neutral evolution, suggesting that demographic events, such us range expansion or population bottleneck, could have determined the observed pattern of molecular diversity. Similarly, given the lack of evidence of a direct relationship between reduction in molecular diversity and increased prevalence of *Wolbachia* in *P*. *spumarius* populations, demographic events are more likely to have determined the pattern of molecular diversity observed in the populations of northern Italy, rather than a *Wolbachia* selective sweep. Coherently, the AMOVA showed that the component of genetic variability associated with the infection status was negligible in *P*. *spumarius*.

One possible reason for the lack of data supporting a selective sweep in Alto Adige, despite the high infection prevalence, is that *Wolbachia* may have spread long ago in *P*. *spumarius* populations of this geographic area, and since then mutations of host mitochondrial DNA have accumulated. Consequently, evidence of selection has now been lost and cannot be detectable by neutrality tests [102, 106]. In *P*. *spumarius*, *Wolbachia* infection is believed to have originated in the NE lineage after the separation of the ancestral lineage into the NE and SW lineages [53]. Therefore, the spread of *Wolbachia* may have occurred in the populations of the Alto Adige following the contact between the two main lineages and the transfer of *Wolbachia* from the NE to the SW lineage. In the event of an ancient infection, together with an increase in mtDNA diversity of the host population, the genetic diversity of *Wolbachia* is also expected to increase due to mutations gained after the last selective sweep, loss of strains and acquisition of new strains by horizontal transfer [102, 112, 113]. Coherently, we found a number of different *Wolbachia* STs infecting *P*. *spumarius* in Alto Adige. In particular, five out of six STs were closely related phylogenetically, suggesting the origin of new STs by mutation of the ST initially spread. Alternatively, NE haplotypes may have invaded the Alto Adige contact zone repeatedly over time by introducing new closely related *Wolbachia* strains and adding diversity in the mtDNA. Indeed, many infected haplotypes of the NE lineage were found in Alto Adige, of which four differed in the *Wolbachia* ST and two shared the same ST. Further, all STs were associated with individuals of the NE lineage and only two of them (ST-538 and ST-539) were shared between the NE and the western-Mediterranean lineage. The most common *Wolbachia* STs in the populations of Alto Adige were ST-539 and ST-538, with 53% and 27% of infected individuals, respectively. This suggests that one of them could be the strain that started the invasion. Finally, because mitochondrial mutation rates are substantially higher than those typically found in bacteria [102], consistent with an ancient infection of *P*. *spumarius*, in Alto Adige we found twice the number of infected haplotypes (10, of which five of the NE lineage and five of the western-Mediterranean lineage) than the number of *Wolbachia* STs (five). The prevalent strain ST-539 was associated with the prevalent haplotype H24 and three H24-derived haplotypes (plus a fourth haplotype, H29, in the Piemonte region; this is the ancestral haplotypes of the western-Mediterranean lineage, from which H24 should be derived). This suggests that *Wolbachia* strains, because inherited vertically, have persisted in haplotypes derived from the one originally infected. Nonetheless, horizontal transfer may have also played a role in diversifying the correlation between haplotype and infection type over time in *P*. *spumarius* populations (see below). The situation reverses in the southernmost regions of northern Italy where *Wolbachia* infections may have been much more recent and still in the initial phase of spread. We found that 43% of the populations sampled in these regions were not infected and the infection rate was highly variable. Further, only two *Wolbachia* STs were found and one of these was ST-539. The prevalent one, ST-549, closely related to ST-539, was distributed within populations of three geographic regions but not in Alto Adige, which is suggestive of its differentiation in this area after the beginning of the invasion, e.g. of ST-539. However, an insufficient size of our sample cannot be excluded as a possible cause of the differences in *Wolbachia* STs composition between the populations of Alto Adige and those of the other regions. Lack of selective sweep in infected populations may be the consequence of *Wolbachia*’s inability to invade host populations because of imperfect maternal transmission and low penetrance or absence of reproductive parasitism (e.g. CI). These factors limit the spread of symbionts in host populations, unless the symbiotic relationship evolves to increase the fitness of female hosts [114, 115]. In this case, *Wolbachia* infections are maintained in insect populations even without appreciable manipulation of host reproduction [10, 116, 117]. These infections may persist by providing direct fitness benefits to the host such as protection against viral pathogens [118, 119], nutritional supplementation [120] or a better exploitation of the insect’s host plant [16, 121]. However, facultative symbionts like *Wolbachia* are not strictly necessary for host survival and reproduction and the benefits they provide are frequently context dependent [122]. For example, bacterial symbionts that confer protection against natural enemies [123] or environmental tolerance [124] are beneficial only if the stress factors are at stake on the insect host population. In their absence, the infection cost exceeds the positive fitness benefits and infected hosts are outcompeted by uninfected ones. Conversely, in environments characterized by fluctuations of the natural enemy populations or temperatures, selection acts to maintain infection polymorphism in the host population [125], and the infected individuals will coexist with the uninfected ones. The phenotype of *Wolbachia* in *P*. *spumarius* is still unknown. We did not find differences between the sex ratio of infected and uninfected populations, ruling out reproductive manipulations inducing female-biased sex ratios. Some authors [53] have suggested that CI might act between the infected individuals of the NE lineage and the uninfected individuals of the SW lineage in the Carpathians contact zone. However, experimental data demonstrating the occurrence of CI in *P*. *spumarius* are not yet available. In the Italian populations of *P*. *spumarius*, the considerable variability of the prevalence of *Wolbachia* infection among the populations in northern Italy, the absence of populations with fixed infection and the lack of evidence of a recent selective sweep, lead us to exclude a strong CI. Should CI occur it is likely weak and not sufficient to ensure successful symbiont spread because of an imperfect maternal transmission and none or limited direct positive effects of *Wolbachia* on host fitness. We did not quantify the transmission rate and the density of *Wolbachia* in *P*. *spumarius*, however we did get some evidence of imperfect maternal transmission. Indeed, infected and uninfected individuals of the same haplotype were present simultaneously within infected populations (i.e., H24 in Alto Adige and H32 in Piemonte and Veneto) and *Wolbachia* did not reached fixation in any of the infected populations. Further, in populations of Alto Adige consisting of admixtures of individuals of the NE and western-Mediterranean lineages, the two lineages did not differentiate based on the infection status, showing the same high infection rate and sharing identical *Wolbachia* STs. A situation opposite to that observed in the Carpathians [53]. Therefore, alternatively to cause CI, *Wolbachia* may be a facultative mutualist of *P*. *spumarius* able to benefit the host in specific environmental contexts, like those at high altitudes in northern Italy, characterized by low temperature regimes and possibly specific host plants and natural enemies. In these conditions, selective forces act to maintain the infected haplotypes, both of the NE and western-Mediterranean lineages, within populations. Conversely, in temperate or Mediterranean climate environments of Italy, the benefit for the host fitness may be less than the cost of the infection and prolonged periods of high temperatures (e.g. >30°C during summer) may affect dramatically on *Wolbachia* density and consequently on transmission efficiency and phenotype expression. Therefore, infected haplotypes may be selected against as they are at a disadvantage compared to uninfected ones. As a result, *Wolbachia* infection is variable and generally low and a diversity of haplotypes is maintained within *P*. *spumarius* populations.

Although the nature of the symbiotic relationship (i.e. reproductive manipulation or facultative mutualism) is still unknown, FISH experiments in this study confirmed a close relationship between *Wolbachia* and *P*. *spumarius*. A genuine vertical transmission of *Wolbachia* through the egg cytoplasm was found, according to a mechanism already highlighted in other studied systems. *Wolbachia* symbionts were localized within the telotrophic ovarioles of *P*. *spumarius*, with the highest concentration in the germarium, where the differentiation of the female germline cells takes place. Through the infection of germline cells, *Wolbachia* symbionts and other reproductive manipulators pass into the oocytes during early oogenesis [126–129]. Moreover, similarly to other insect species infected by endosymbiotic bacteria [126, 129, 130], *Wolbachia* concentrated at the posterior pole of the oocyte during late oogenesis of *P*. *spumarius*. During embryogenesis, the posterior pole of the egg is the site where the germ cells differentiate, therefore the concentration at the posterior pole is a mechanism that increases the chance that symbionts are integrated into the germ cells and are transmitted to the progeny of the infected female [131–133]. *Wolbachia* did not localize within bacteriocytes of *P*. *spumarius* and their way of transovarial transmission differed from that of primary symbionts. These one, as previously observed in other insects [134–137], after being released from the bacteriocytes, do not infect the germarium like *Wolbachia* does, but enter the posterior pole of the developing oocyte and accumulate in the ooplasm forming a characteristic mass (symbiont ball). Our FISH experiments also revealed the localization of *Wolbachia* in the salivary glands of *P*. *spumarius*. This finding was also supported by PCR that revealed a positive signal for *Wolbachia* when DNA was extracted from the head of infected individuals. The localization of facultative bacterial symbionts, including *Wolbachia*, in the salivary glands has been observed in other species of sap-feeding insects in Auchenorrhyncha [138–141]. In one of the leafhopper species investigated, *Scaphoideus titanus* Ball [138, 140], facultative bacterial symbionts are released from the salivary glands during feeding and are subsequently acquired by other insects co-feeding upon the host plant. In addition, plant-mediated horizontal transmission of *Wolbachia* (and *Rickettsia* [142]), followed by vertical transmission to progeny, has been shown in whiteflies feeding in the host phloem [143]. *Wolbachia* infection of *P*. *spumarius* salivary glands, together with the non-specific association between *Wolbachia* ST and host haplotype, suggest that intraspecific horizontal transmission may occur during feeding into the xylem. After the injection of saliva containing *Wolbachia* by infected individuals, the symbionts may be acquired by other individuals who feed on the same plant. Coherently, we found that three *Wolbachia* STs were each associated with multiple haplotypes belonging to different mitochondrial lineages. Moreover, suggestive of interspecific horizontal transmission, was that *Wolbachia* ST-537 of *P*. *spumarius* was closely related with ST-217 found both in the leafhopper *M*. *fascifrons* and in the spittlebug *Neophilaenus campestris* (Fallén) [54]. The latter species commonly shares host plants with *P*. *spumarius* in both agricultural and natural environments [144, 145]. Although leafhoppers are phloem-feeders and spittlebugs are xylem-feeders, feeding guilds are not strict categories and, especially among vascular-feeder leafhoppers, the distinction between the phloem-feeding and xylem-feeding guilds is blurred [146], thus possibly facilitating the horizontal transmission of microbial symbionts. The acquisition of *Wolbachia* strains in *P*. *spumarius* from multiple unrelated donor species (e.g., chrysomelids, drosophilids, mites, vespids, weevils, whiteflies) was first suggested by [53] and would appear to be common in other species of spittlebugs [54, 55]. Horizontal transmission may be a key factor in the infection dynamic and maintenance of heritable microbial symbionts, especially if they cause weak reproductive manipulations [143, 147]. Acquisition and subsequent transmission of bacterial symbionts through feeding on plants may complement vertical transmission, favoring the spread and persistence of symbionts within the insect host populations [148–150]. In *P*. *spumarius*, intra- and inter-specific horizontal transmission via plant may be a rampant process contributing to the maintenance of *Wolbachia* within the infected populations of northern Italy, but probably not sufficient to compensate for the loss of infections, due to imperfect maternal transmission and environmental curing.

The suppression of vector populations is pivotal for the control of vector-borne diseases, and the system *P*. *spumarius—X*. *fastidiosa* is no exception [24, 151]. Mechanical control of nymphs and insecticides-based control of pre-infective adults of *P*. *spumarius* are only partially effective and the use of chemicals is associated to unwanted side effects. *Wolbachia*-based biological control methods could complement existing current tactics in reducing the populations of *P*. *spumarius* and therefore the spread and incidence of *X*. *fastidiosa*. The IIT strategy [21, 22] could be an option in conditions as those of the expansion area of *X*. *fastidiosa* subspecies *pauca* in southern Italy [39, 40]. There, the populations of *P*. *spumarius*, almost exclusively of the eastern-Mediterranean lineages, do not harbor *Wolbachia* and could be adversely affected by the inundative release of *Wolbachia*-infected males (e.g., of the western-Mediterranean lineages from northern Italy). Crossing experiments are imperative to verify whether CI actually occurs and whether it is sufficiently strong to think about an application of IIT. Alternatively, the population replacement approach [22] may be difficult to apply in southern Italy. In this area, based on our results, environmental factors (e.g. long periods of hot and dry climate) and the genetic background of *P*. *spumarius* do not seem to allow the spread of *Wolbachia* infection. Moreover, for the application of the *Wolbachia*-based vector control, it is important to evaluate if and how *Wolbachia* can affect the transmission of *X*. *fastidiosa*. A prerequisite for the applicability of IIT method should be a low ability of released males to transmit *X*. *fastidiosa* to avoid that the benefit of vector population reduction is outweighed by a greater spread of the pathogen soon after the release of incompatible males. Bacterial endosymbionts are key factors regulating the interactions between vectors and phloem-limited phytopathogenic bacteria, having antagonistic or beneficial effects on plant pathogens circulating in the insect body [152]. Instead, the impact of bacterial endosymbionts on *X*. *fastidiosa* is expected to be less evident due to the absence of an intimate relationship between vector and pathogen. *Xylella fastidiosa* does not interact with vector’s endogenous factors as it colonizes the surface of precibarium and cibarium regions of the vector’s foregut and is transmitted soon after the acquisition in a propagative and non-circulative way [153]. Indeed, the transmission efficiency of *X*. *fastidiosa* does not seem to vary within a vector species (while there are differences between the vector species) [154]. We showed that *Wolbachia* symbionts are able to localize within the salivary glands of *P*. *spumarius*. Watery saliva injected by insect vectors into plants seems to play a role during the inoculation of *X*. *fastidiosa* [155, 156]. Whether *Wolbachia* symbionts of *P*. *spumarius* can somehow interfere with the inoculation of *X*. *fastidiosa* through antagonistic compounds produced in the salivary glands and released into the host’s saliva is just an intriguing speculation maybe worthy to be investigated.

## Supporting information

S1 Fig Median joining haplotype network obtained from COI gene sequences of *Philaenus spumarius* collected worldwide. Circle sizes are proportional to haplotype frequency. Colors correspond to the country of origin of the haplotype. Small black dot vertices represent missing or unsampled haplotypes. Diamonds on branches represent the number of mutations. No diamond on branches means one mutation.
(PDF)

S2 Fig Frequency of mitochondrial lineages of *Philaenus spumarius* and *Wolbachia* infection rate in the three macro-areas of Italy. Histograms show the frequency of the three mitochondrial lineages each one represented by a different color (green for the eastern-Mediterranean, yellow for the western-Mediterranean and white for the eastern lineage). Cakes show the *Wolbachia* infection rate in northern (red), central (grey) and southern (green) Italy. The percentage of infected individuals is the slice of cake colored with checkered texture. Similarly, regions with populations of *P*. *spumarius* infected by *Wolbachia* are colored with checkered texture. The big cakes represent the *Wolbachia* infection rate calculated on the total number of individuals sampled in each of the three macro-areas. The small cakes represent the *Wolbachia* infection rate calculated on the individuals of each mitochondrial lineage. Individuals of *P*. *spumarius* from the colored regions were analyzed to assess the distribution of mitochondrial lineages. The populations analyzed for *Wolbachia* infection were sampled in checkered, dark grey and dark green colored regions. White regions have not been sampled. The map of Italy by Sinigagl (https://commons.wikimedia.org/wiki/File:Italy_template_blank.png) is licensed under CC BY-SA 3.0; it is similar but not identical (it differs for the colored regions) to the original map and is therefore for illustrative purposes only.
(PDF)

S3 Fig Bayesian phylogenetic tree for single MLST genes of *Wolbachia*. (A) *gatB* gene. (B) *coxA* gene. (C) *hpcA* gene. (D) *ftsZ* gene. (E) *fbpA* gene. Alleles found in *Philaenus spumarius* and sequenced in this work are shown in red, while alleles sequenced by [53] are in black. Alleles of reference *Wolbachia* STs were imported from the MLST database and are indicated on the tree with the name of the host species, the number of the ST and the supergroup letter.
(PDF)

S4 Fig Maximum likelihood phylogenetic tree for concatenated MLST gene sequences of *Wolbachia*. STs found in *Philaenus spumarius* and sequenced in this work are shown in bold red. Reference *Wolbachia* STs were imported from the MLST database and are indicated on the tree with the name of the host species and the number of the ST. *Wolbachia* strains of *P*. *spumarius* by [53] are reported in bold black and with the original strain number (ST number not available in the MLST database). Based on the information reported in the MLST database for the reference strains, the letters on the right indicate the *Wolbachia* supergroups.
(PDF)

S5 Fig Maximum likelihood phylogenetic tree for single MLST genes of *Wolbachia*. (A) *gatB* gene. (B) *coxA* gene. (C) *hpcA* gene. (D) *ftsZ* gene. (E) *fbpA* gene. Alleles found in *Philaenus spumarius* and sequenced in this work are shown in red, while alleles sequenced by [53] are in black. Alleles of reference *Wolbachia* STs were imported from the MLST database and are indicated on the tree with the name of the host species, the number of the ST and the supergroup letter.
(PDF)

S6 Fig Neighbour-net phylogenetic network based on concatenated MLST gene sequences of *Wolbachia* found in *Philaenus spumarius*. *Wolbachia* STs sequenced in this work are shown in red (IT means Italy). *Wolbachia* strains of *P*. *spumarius* by [53] are shown in green with the original strain number preceded by the acronym of the Country of origin.
(PDF)

S7 Fig Neighbour-net phylogenetic network for single MLST genes of *Wolbachia*. (A) *gatB* gene. (B) *coxA* gene. (C) *hpcA* gene. (D) *ftsZ* gene. (E) *fbpA* gene. Alleles found in *Philaenus spumarius* and sequenced in this work are shown in red, while alleles sequenced by [53] are shown in green. Reference *Wolbachia* STs were imported from the MLST database and are indicated with the name of the host species, the number of the ST and the supergroup letter.
(PDF)

S8 Fig Fluorescence in-situ hybridization of *Philaenus spumarius* ovarioles. Samples stained by (A) a *Wolbachia*-specific probe (bright red) and (B) a universal bacterial probe (bright green), contrasting with the autofluorescence background. (C) Merged image of panels A and B, and nuclei of the host cells (blue) counterstained with DAPI, showing *Wolbachia* bacteria (yellow) stained with both probes and the formation of the primary symbiont ball (green). (D) Developing oocyte of panel C showing the distribution of *Wolbachia* within the ooplasm. Bars, 100 μm.
(PDF)

S9 Fig Fluorescence in-situ hybridization of *Philaenus spumarius*. Samples simultaneously stained by a *Wolbachia*-specific probe (red) and a universal bacterial probe (green). (A) Bacteriomes of a *Wolbachia*-infected individual showing the green signal of the universal probe but no red signal of the *Wolbachia* probe. (B-D) Ovarioles and developing oocytes of *Wolbachia*-uninfected individuals with the universal probe showing the primary symbiont penetration into the oocyte and the symbiont ball. (E-F) Salivary glands of *Wolbachia*-uninfected individuals showing no signal of bacteria and the autofluorescence background. Bars, 100 μm.
(PDF)

S1 Table Populations of *Philaenus spumarius* sampled in Italy. (PDF)

S2 Table Haplotypes of the *Philaenus spumarius COI* gene and their association with *Wolbachia*. (PDF)

S3 Table Sex ratio in the populations of *Philaenus spumarius* (A) and statistical analysis (G-test of independence) (B).
(PDF)

S4 Table Molecular diversity parameters of *Philaenus spumarius* and neutrality tests for the overall data set and the populations grouped by region of origin. *n*, number of haplotypes; *S*, segregating sites; *Hd*, haplotype diversity; π, nucleotide diversity; *k*, mean number of pairwise differences; ^**w**^, group including *Wolbachia*-infected populations; ^NE^, without haplotypes of the north-eastern lineage.
(PDF)

S5 Table Molecular diversity parameters and neutrality tests for the haplotypes of *Philaenus spumarius* grouped by mitochondrial lineage. *n*, number of haplotypes; *S*, segregating sites; *Hd*, haplotype diversity; *π*, nucleotide diversity; *k*, mean number of pairwise differences.
(PDF)

S6 Table Molecular diversity parameters and neutrality tests for the individuals of *Philaenus spumarius* grouped by the status of *Wolbachia* infection. *n*, number of haplotypes; *S*, segregating sites; *Hd*, haplotype diversity; *π*, nucleotide diversity; *k*, mean number of pairwise differences.
(PDF)

S7 Table Analysis of molecular variance (AMOVA) based on *COI* gene sequences of 27 populations of *Philaenus spumarius*. (PDF)

S8 Table Analysis of molecular variance (AMOVA) run on *COI* gene haplotypes of *Philaenus spumarius* individuals grouped according their infection status (infected or uninfected). (PDF)

S9 Table Analysis of molecular variance (AMOVA) run on *COI* gene haplotypes of individuals of the western-Mediterranean lineage of *Philaenus spumarius* grouped according their infection status (infected or uninfected). (PDF)

S10 Table *Wolbachia* sequence types (ST) of *Philaenus spumarius* and their association with host population and haplotype. (PDF)

S11 Table Distribution of mitochondrial lineages of *Philaenus spumarius* among the geographic regions of Italy. (PDF)

S12 Table Genetic divergence (uncorrected p-distance) between and within phylogeographic lineages of *Philaenus spumarius* calculated on COI gene haplotypes sampled in Italy. (XLSX)

S13 Table Genetic divergence (uncorrected p-distance) between MLST gene sequences of *Wolbachia*. *Wolbachia* STs of *Philaenus spumarius* sequenced in this study are indicated in bold red. *Wolbachia* strains of *P*. *spumarius* sequenced by [53] are indicated in bold black, among which those in italics are from Italy. Reference STs from the MLST Database are indicated in normal style.
(XLSX)
