# Supplementary figures and images for: Wolbachia infection and genetic diversity of Italian populations of Philaenus spumarius, the main vector of Xylella fastidiosa in Europe
Source: PLoS One. 2022 Aug 29;17(8):e0272028. doi: 10.1371/journal.pone.0272028 (PMC9423658; doi:10.1371/journal.pone.0272028)

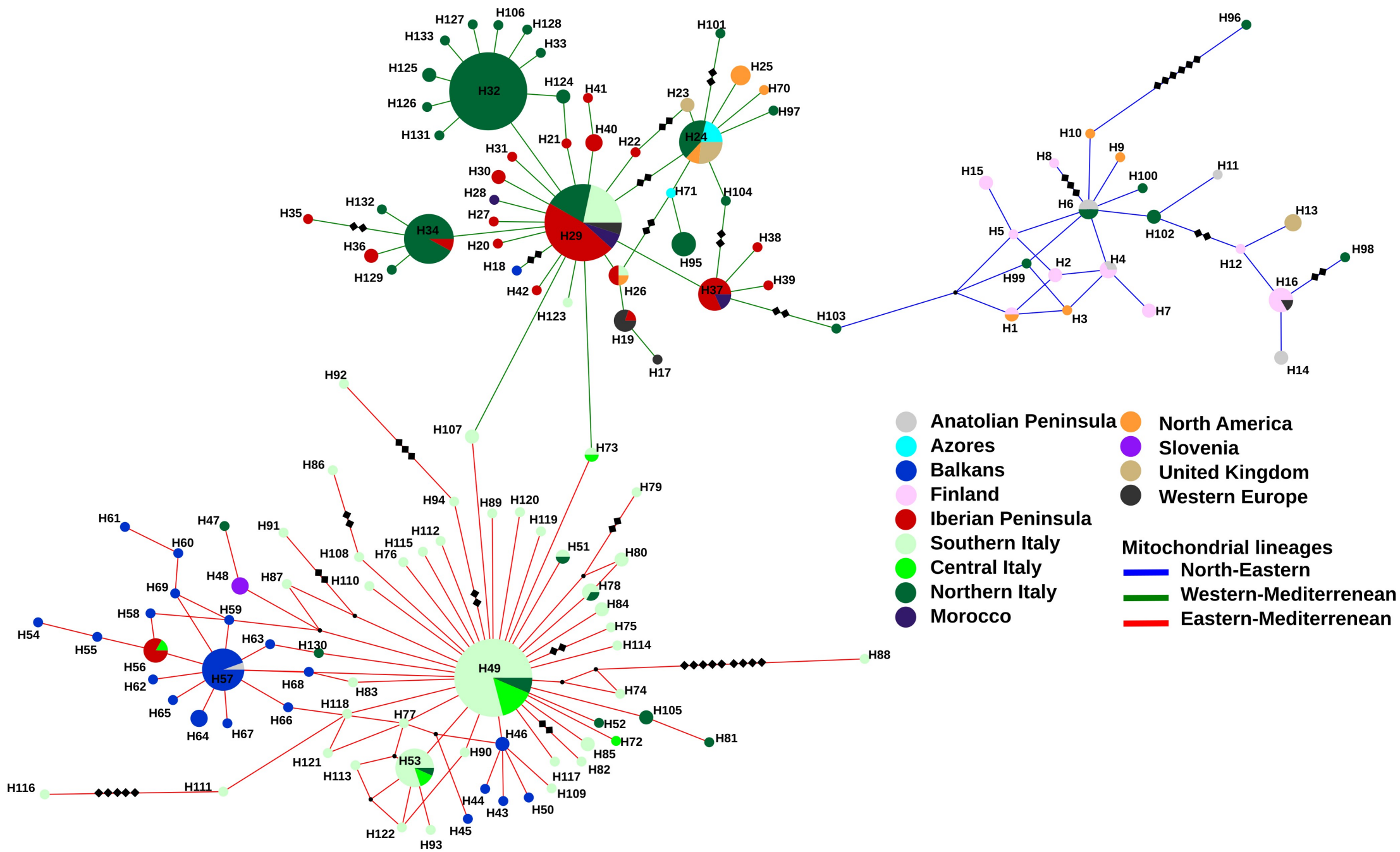

Supplement: S1 Fig — Circle sizes are proportional to haplotype frequency. Colors correspond to the country of origin of the haplotype. Small black dot vertices represent missing or unsampled haplotypes. Diamonds on branches represent the number of mutations. No diamond on branches means one mutation. (PDF) [file pone.0272028.s001.pdf]

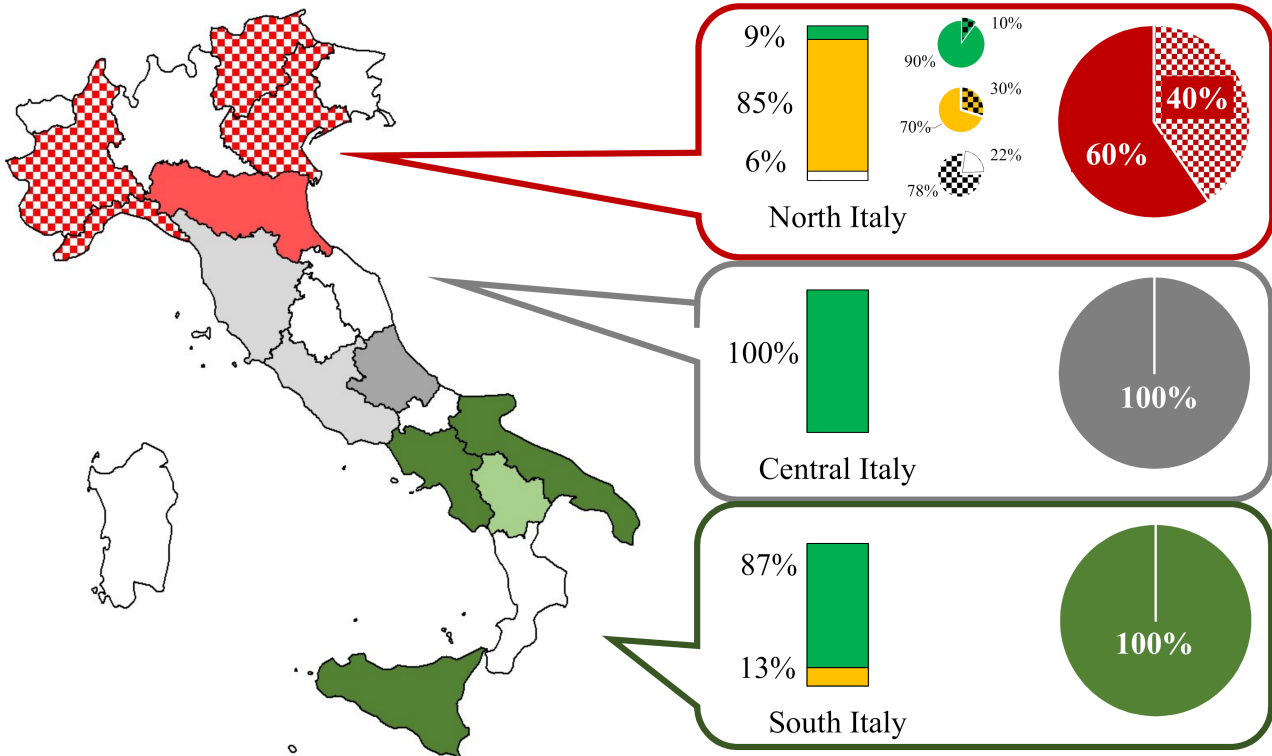

Mitochondrial lineages: ■ Eastern-Mediterranean

■ Western-Mediterranean

North-Eastern

Supplement: S2 Fig — Histograms show the frequency of the three mitochondrial lineages each one represented by a different color (green for the eastern-Mediterranean, yellow for the western-Mediterranean and white for the eastern lineage). Cakes show the Wolbachia infection rate in northern (red), central (grey) and southern (green) Italy. The percentage of infected individuals is the slice of cake colored with checkered texture. Similarly, regions with populations of P. spumarius infected by Wolbachia are colored with checkered texture. The big cakes represent the Wolbachia infection rate calculated on the total number of individuals sampled in each of the three macro-areas. The small cakes represent the Wolbachia infection rate calculated on the individuals of each mitochondrial lineage. Individuals of P. spumarius from the colored regions were analyzed to assess the distribution of mitochondrial lineages. The populations analyzed for Wolbachia infection were sampled in checkered, dark grey and dark green colored regions. White regions have not been sampled. The map of Italy by Sinigagl (https://commons.wikimedia.org/wiki/File:Italy_template_blank.png) is licensed under CC BY-SA 3.0; it is similar but not identical (it differs for the colored regions) to the original map and is therefore for illustrative purposes only. (PDF) [file pone.0272028.s002.pdf]

(A) *gatB* gene

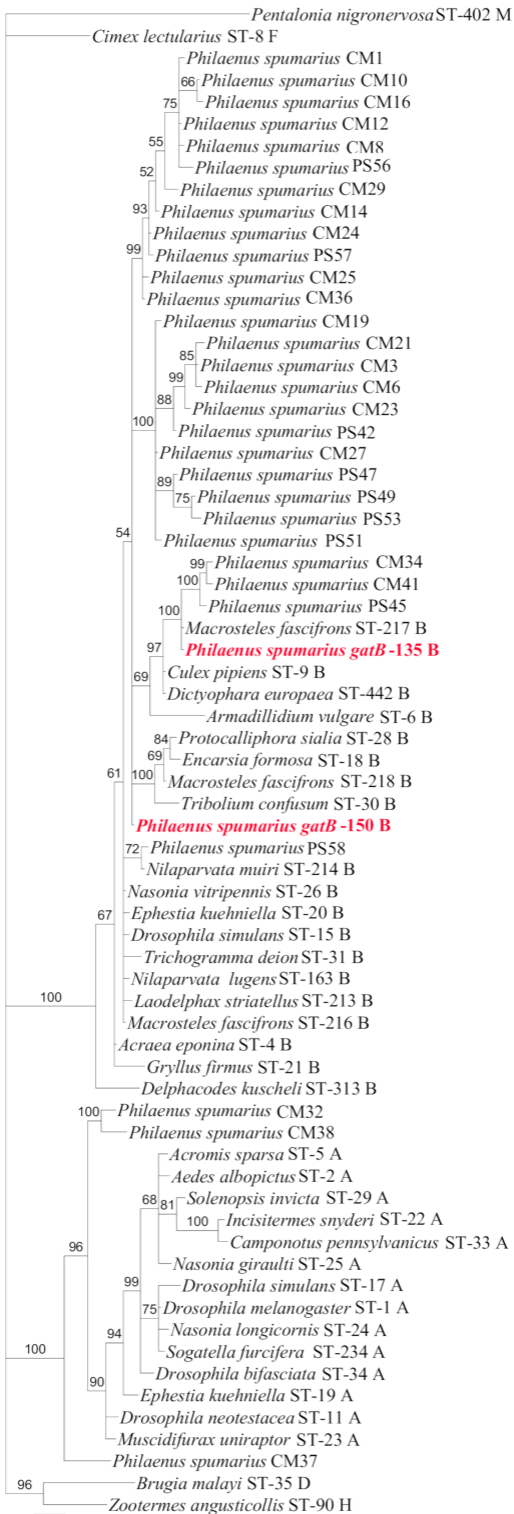

(B) *coxA* gene

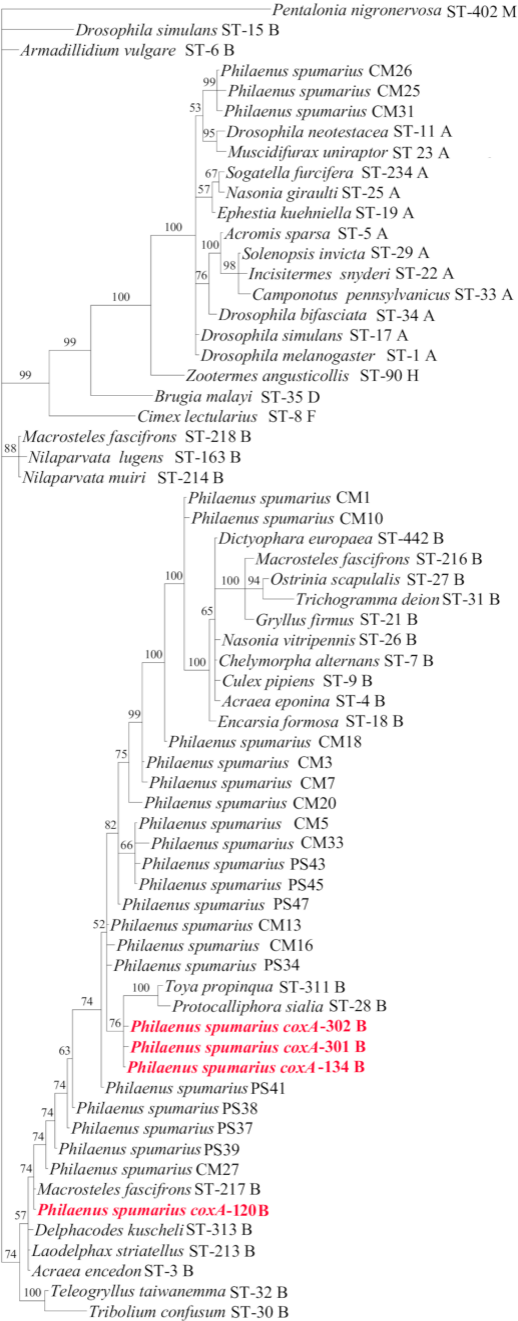

(C) *hcpA* gene

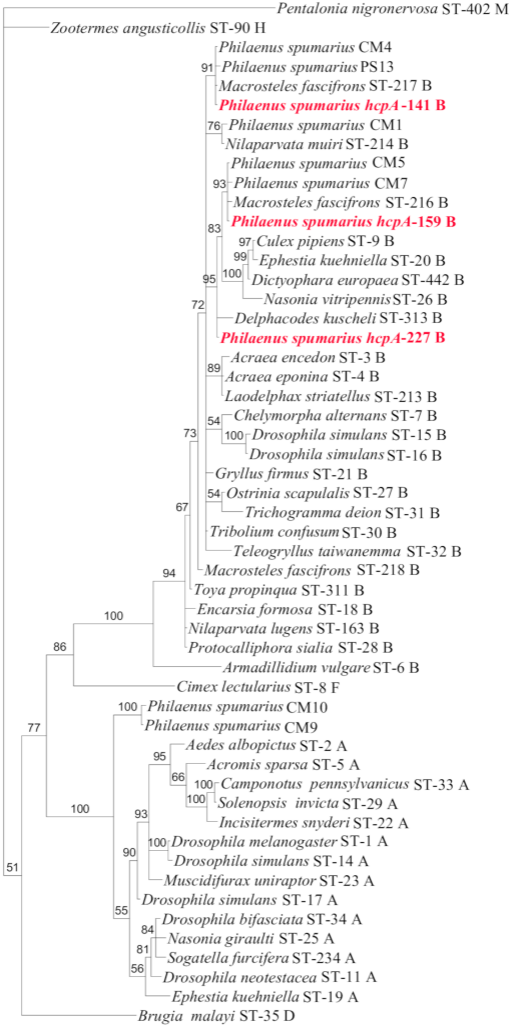

(D) *ftsZ* gene

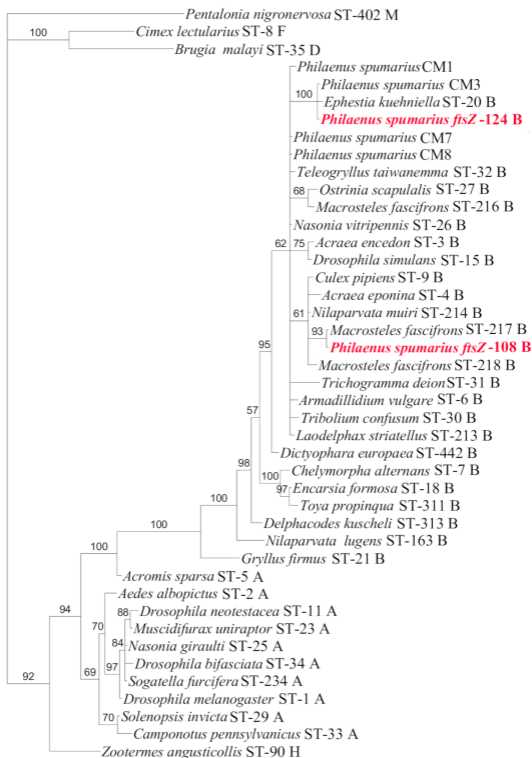

(E) *fbpA* gene

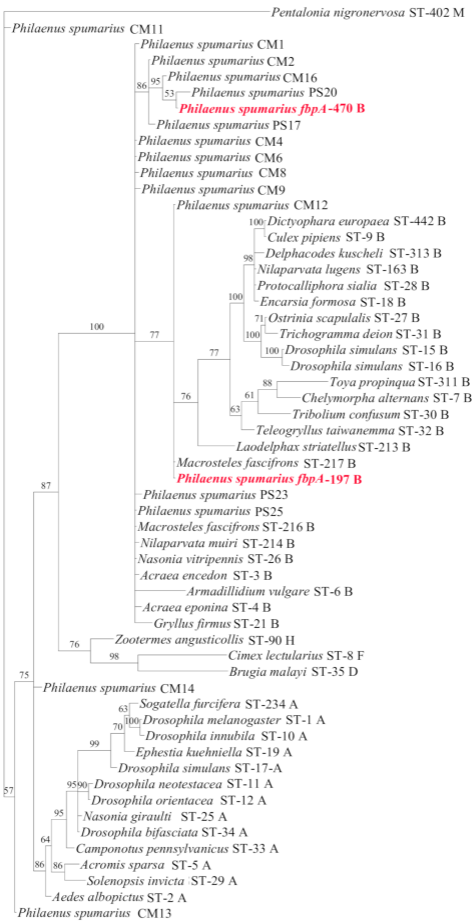

Supplement: S3 Fig — (A) gatB gene. (B) coxA gene. (C) hpcA gene. (D) ftsZ gene. (E) fbpA gene. Alleles found in Philaenus spumarius and sequenced in this work are shown in red, while alleles sequenced by [53] are in black. Alleles of reference Wolbachia STs were imported from the MLST database and are indicated on the tree with the name of the host species, the number of the ST and the supergroup letter. (PDF) [file pone.0272028.s003.pdf]

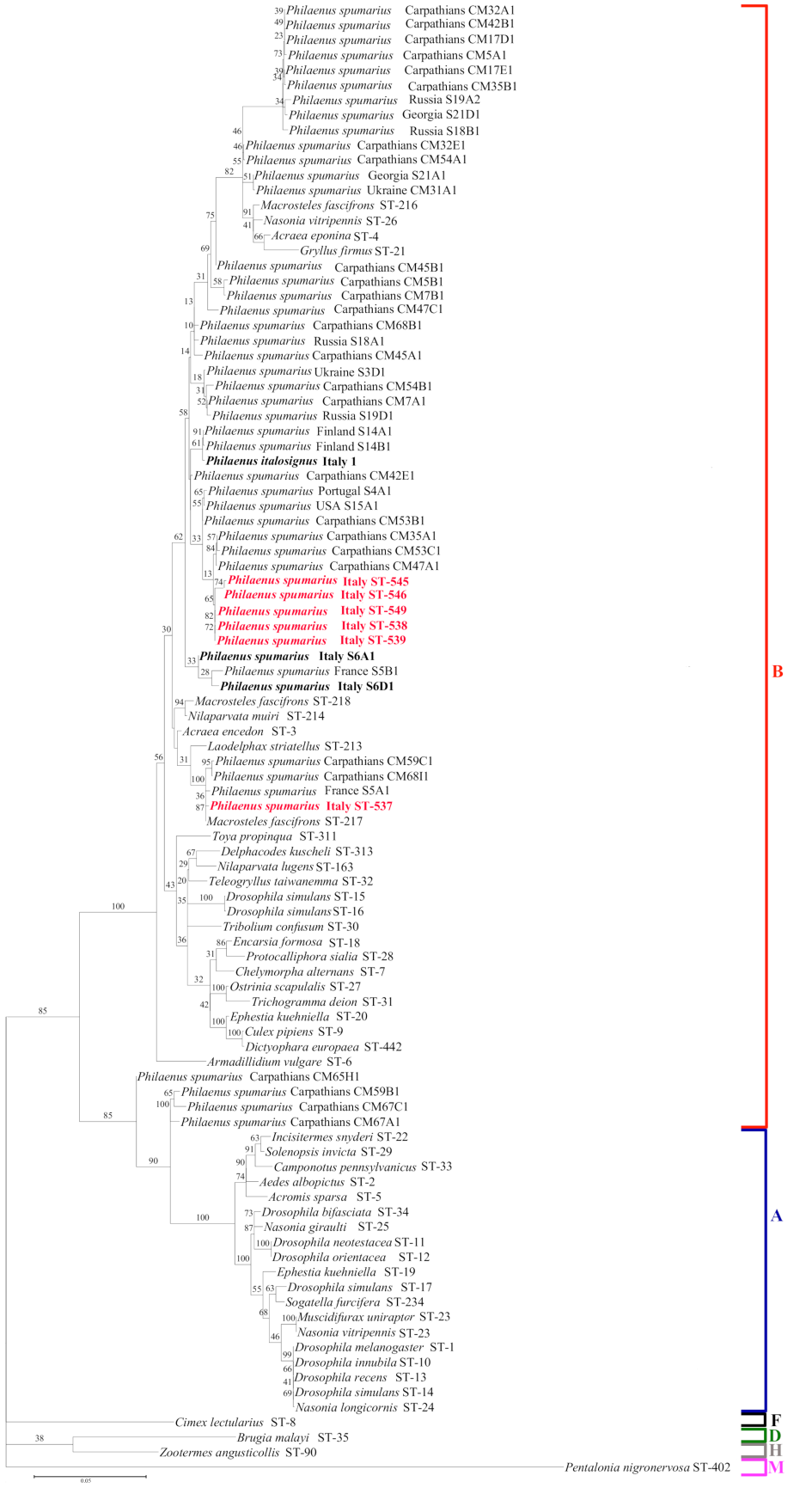

Supplement: S4 Fig — STs found in Philaenus spumarius and sequenced in this work are shown in bold red. Reference Wolbachia STs were imported from the MLST database and are indicated on the tree with the name of the host species and the number of the ST. Wolbachia strains of P. spumarius by [53] are reported in bold black and with the original strain number (ST number not available in the MLST database). Based on the information reported in the MLST database for the reference strains, the letters on the right indicate the Wolbachia supergroups. (PDF) [file pone.0272028.s004.pdf]

(A) *gatB* gene

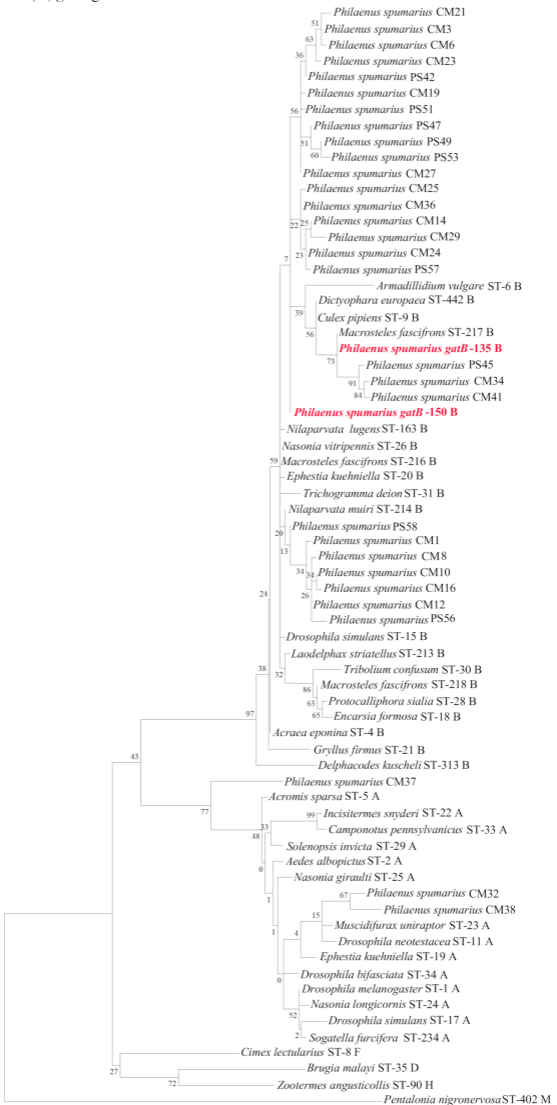

0.05

(B) *coxA* gene

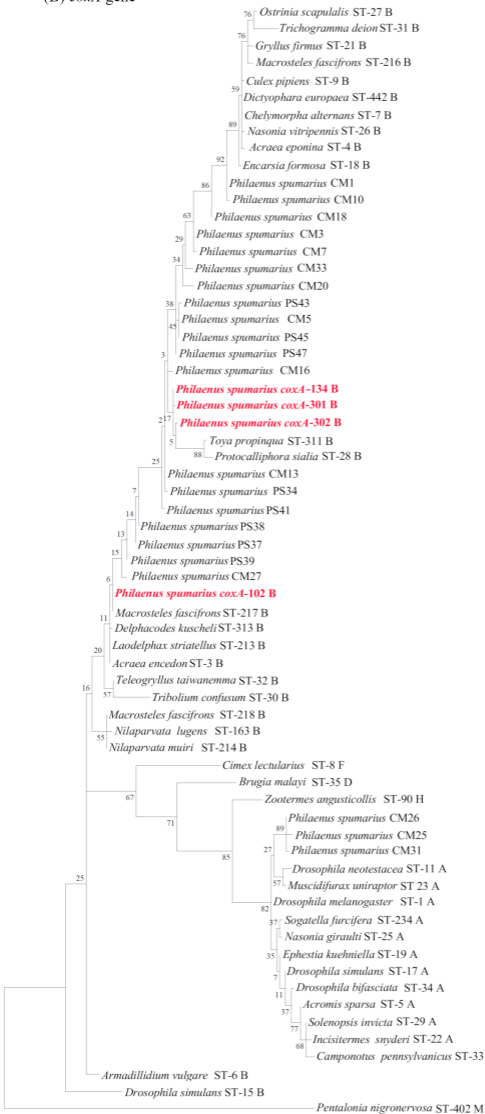

0.05

(C) *hcpA* gene

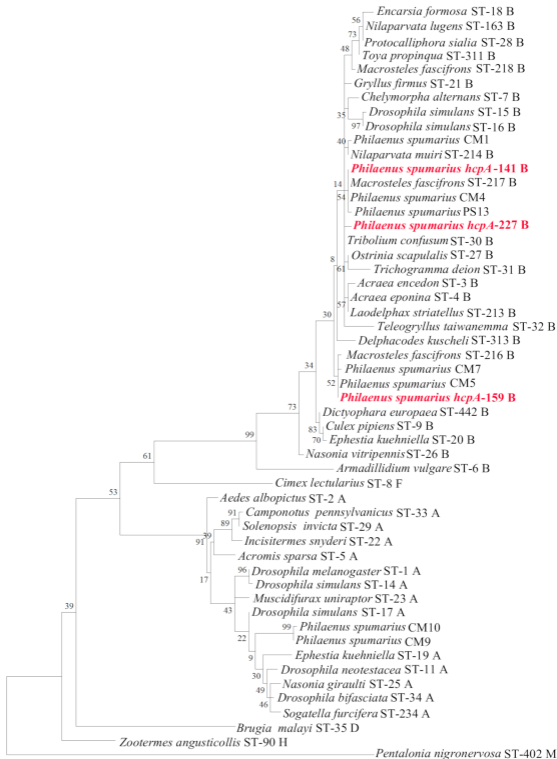

0.05

(D) *ftsZ* gene

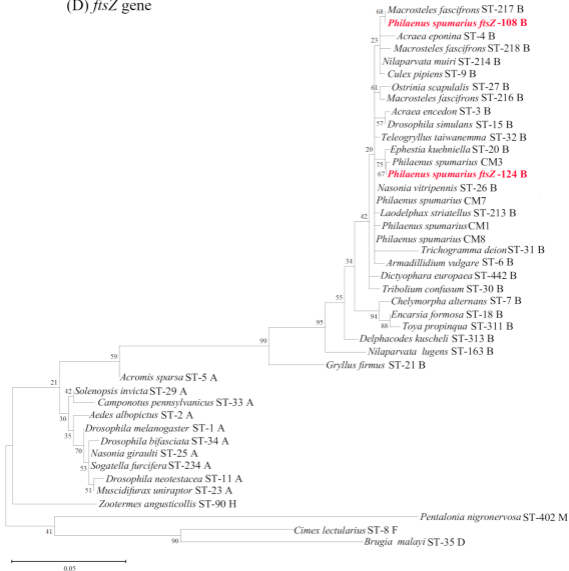

(E) *fbpA* gene

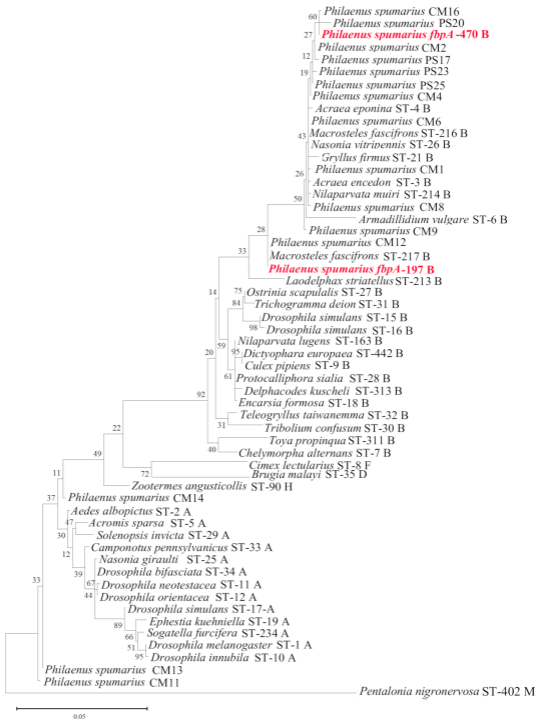

Supplement: S5 Fig — (A) gatB gene. (B) coxA gene. (C) hpcA gene. (D) ftsZ gene. (E) fbpA gene. Alleles found in Philaenus spumarius and sequenced in this work are shown in red, while alleles sequenced by [53] are in black. Alleles of reference Wolbachia STs were imported from the MLST database and are indicated on the tree with the name of the host species, the number of the ST and the supergroup letter. (PDF) [file pone.0272028.s005.pdf]

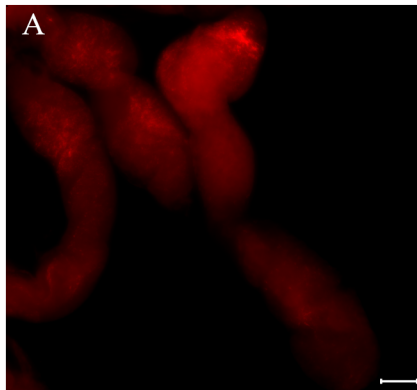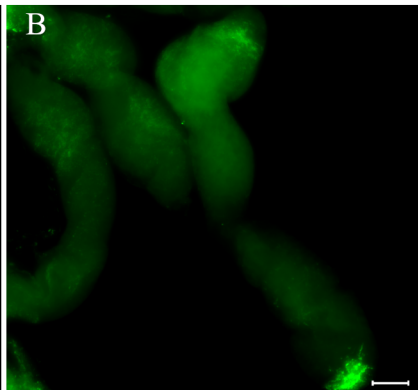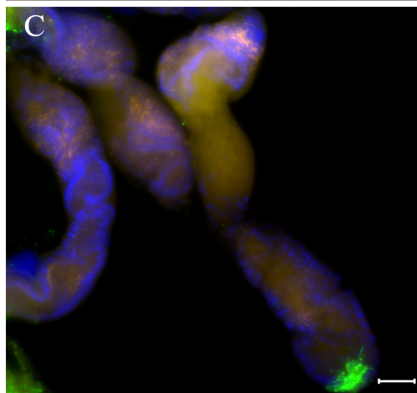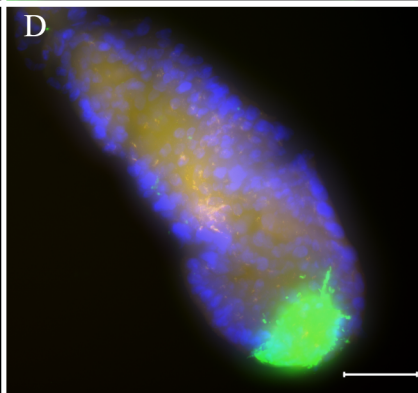

Supplement: S6 Fig — Wolbachia STs sequenced in this work are shown in red (IT means Italy). Wolbachia strains of P. spumarius by [53] are shown in green with the original strain number preceded by the acronym of the Country of origin. (PDF) [file pone.0272028.s006.pdf]

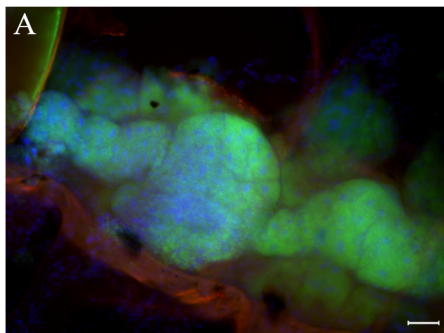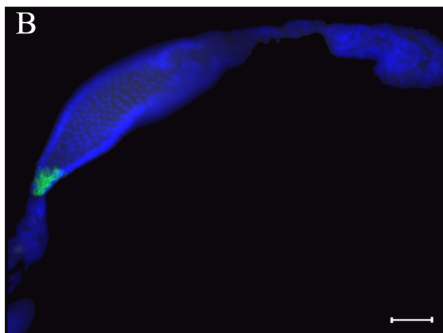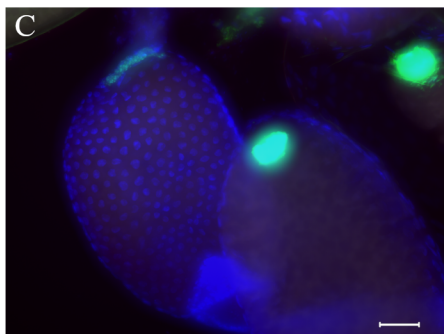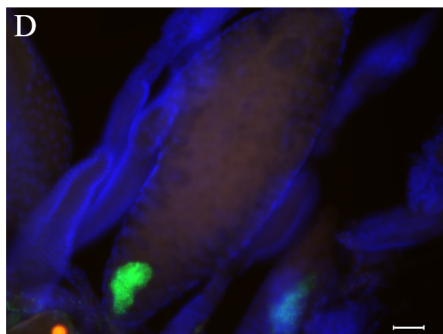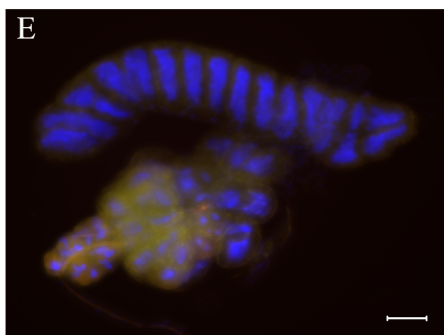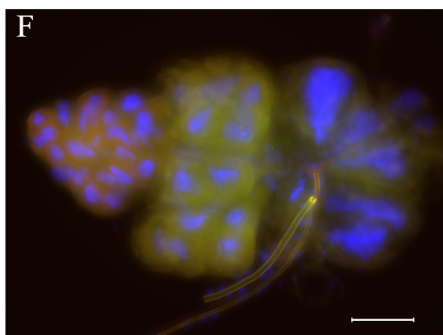

Supplement: S7 Fig — (A) gatB gene. (B) coxA gene. (C) hpcA gene. (D) ftsZ gene. (E) fbpA gene. Alleles found in Philaenus spumarius and sequenced in this work are shown in red, while alleles sequenced by [53] are shown in green. Reference Wolbachia STs were imported from the MLST database and are indicated with the name of the host species, the number of the ST and the supergroup letter. (PDF) [file pone.0272028.s007.pdf]
