# Supplementary material for: Wolbachia infection and genetic diversity of Italian populations of Philaenus spumarius, the main vector of Xylella fastidiosa in Europe
Source: PLoS One. 2022 Aug 29;17(8):e0272028. doi: 10.1371/journal.pone.0272028 (PMC9423658; doi:10.1371/journal.pone.0272028)
Supplement: S1 Table — (PDF) [file pone.0272028.s010.pdf]

S1 Table. Populations of *Philaenus spumarius* sampled in Italy

Population acronym	Location	Province	Region	Coordinates	Altitude (m asl)	Sampling date (year.months)	Habitat	Individuals screened for *Wolbachia* infection	Individuals sequenced for the *COI* gene	Analysis of molecular diversity
**Northern Italy**										
Aa1	Compaccio	Bolzano	Alto Adige	46.54115N, 11.61704E	1848	2018.07-09	meadows	47	8	Yes
Aa2	Castelrotto	Bolzano	Alto Adige	46.56417N, 11.55132E	1000	2018.07-09	meadows	58	13	Yes
Aa3	San Michele	Bolzano	Alto Adige	46.57830N, 11.60203E	1283	2018.07-09	meadows	40	10	Yes
Li1	Finale Ligure	Savona	Liguria	44.18089N, 8.363513E	257	2016.05	herbaceous vegetation near olive grove	4	2	No
Li2	Ortovero	Savona	Liguria	44.054465N, 8.09617E	160	2016.07	herbaceous vegetation near olive groves	0	1	No
Pi1	Asti	Asti	Piemonte	44.92192N, 8.19570E	185	2018.07	herbaceous vegetation near vineyeards	15	12	Yes
Pi3	Venaria	Torino	Piemonte	45.14770N, 7.597928E	290	2017.10	meadows	13	12	Yes
Pi4	Grugliasco	Torino	Piemonte	45.05244N, 7.590030E	280	2016.07	meadows	0	1	No
Pi5	Castellero	Asti	Piemonte	44.915803N, 8.067924E	200	2017.09	herbaceous vegetation near hazelnuts	7	4	Yes
Pi6	Castellamonte	Torino	Piemonte	45.391126N, 7.683194E	379	2017.10	herbaceous vegetation near vineyeards	6	6	Yes
Pi7	Fossano	Cuneo	Piemonte	44.532195N, 7.745778E	350	2017.11	herbaceous vegetation near hazelnuts	5	5	Yes
Ve1	Thiene	Vicenza	Veneto	45.677533N, 11.511060E	89	2015.09	wine grape plants	5	5	Yes

Ve3	Barbarano Vicentino	Vicenza	Veneto	45.409000N, 11.546725E	35	2017.08	wine grape plants	11	11	Yes
Ve4	Bardolino	Verona	Veneto	45.541489N, 10.732594E	115	2017.09	olive trees	11	12	Yes
Ve5	Belvedere	Vicenza	Veneto	45.685838N, 11.771236E	70	2015.08	meadows	5	5	Yes
Ve6	Lazise	Verona	Veneto	45.508711N, 10.738042E	94	2017.08	olive trees	11	11	Yes
Ve7	Montegrotto Terme	Padova	Veneto	45.315586N, 11.772342E	114	2017.08	olive trees	6	6	Yes
Ve8	Montecchio Precalcino	Vicenza	Veneto	45.654492N, 11.557116E	70	2015.09	meadow near vineyards	5	5	Yes
Ve9	Bussolengo	Verona	Veneto	45.447969N, 10.862103E	105	2017.08	olive trees	13	9	Yes
**Central Italy**										
Ab1	Colonnella	Teramo	Abruzzo	42.86796N, 13.84821E	190	2017.08	olive trees	13	4	Yes
Ab2	Torino di Sangro	Chieti	Abruzzo	42.20542N, 14.53579E	65	2017.08	olive trees	12	6	Yes
Ab3	Pineto	Teramo	Abruzzo	42.60543N, 14.04148E	50	2017.08	olive trees	11	0	No
Ab4	Spoltore	Pescara	Abruzzo	42.22732N, 14.42341E	240	2017.08	olive trees	13	0	No
**Southern Italy**										
Ba1	Potenza	Potenza	Basilicata	40.64009N, 15.79847E	712	2016.04	meadow	0	1	No
Ca1	Montesarchio	Benevento	Campania	41.08618N, 14.65490E	548	2017.05	herbaceous vegetation near olive groves	17	9	Yes
Ca2	Giffoni Valle Piana	Salerno	Campania	40.75389N, 14.92138E	432	2017.04-10	herbaceous vegetation near hazelnuts	18	6	Yes
Ca3	Montevergine	Avellino	Campania	40.93736N, 14.71813E	1431	2017.05-09	meadows	17	10	Yes
Ca4	Vico Equense	Napoli	Campania	40.65813N, 14.46419E	563	2017.05	herbaceous vegetation near orchards	20	6	Yes
Ca5	Bellizzi	Salerno	Campania	40.64888N,	81	2017.04	herbaceous	20	0	No

				14.96138E			vegetation near hazelnuts			
Ca6	Giffoni Valle Piana	Salerno	Campania	40.70788N, 14.95645E	205	2017.04	herbaceous vegetation near hazelnuts	20	0	No
Ca7	Giffoni Valle Piana	Salerno	Campania	40.76515N, 14.91410E	509	2017.04	herbaceous vegetation near hazelnuts	5	0	No
Ca8	Giffoni Valle Piana	Salerno	Campania	40.70674N, 14.94007E	153	2017.04-10	herbaceous vegetation near hazelnuts	30	0	No
Ca9	Castellamare di Stabia	Napoli	Campania	40.68001N, 14.49622E	289	2017.05	herbaceous vegetation near vineyards	14	0	No
Ca10	Vico Equense	Napoli	Campania	40.67025N, 14.47008E	1027	2017.05	meadows	5	0	No
Ca11	Vico Equense	Napoli	Campania	40.66973N, 14.45877E	729	2017.05	meadows	3	0	No
Ca12	Vico Equense	Napoli	Campania	40.66432N, 14.43596E	196	2017.05	herbaceous vegetation near olive groves	13	0	No
Ca13	Torre le Nocelle	Avellino	Campania	41.02830N, 14.96190E	420	2017.05	herbaceous vegetation near vineyards	18	0	No
Ca14	Bocca della Selva	Benevento	Campania	41.37605N, 14.51044E	1313	2017.06-10	meadows	32	0	No
Ca15	Montesarchio	Benevento	Campania	41.07197N, 14.65693E	397	2017.05-09	herbaceous vegetation near olive groves	19	0	No
Ca16	Tocco Caudio	Benevento	Campania	41.10347N, 14.63497E	676	2017.05	herbaceous vegetation near olive groves	20	0	No
Ca17	Frasso Telesino	Benevento	Campania	41.13907N, 14.51867E	309	2017.05-10	herbaceous vegetation near olive groves	16	0	No
Ca18	San Mango sul Calore	Avellino	Campania	40.95844N, 14.97031E	500	2017.05	herbaceous vegetation near olive groves	10	0	No
Ca19	San Martino	Avellino	Campania	41.01755N,	366	2017.05-10	herbaceous	21	0	No

	Valle Caudina			14.66333E			vegetation near olive groves			
Ca20	Pannarano	Benevento	Campania	41.00475N, 14.70121E	406	2017.05	herbaceous vegetation near olive groves	18	0	No
Ca21	Pannarano	Benevento	Campania	41.02275N, 14.70321E	277	2017.10	herbaceous vegetation near olive groves	7	0	No
Ca22	Portici	Napoli	Campania	40.81517N, 14.35143E	91	2017.09	herbaceous vegetation near olive groves	4	0	No
Pu1	Alliste	Lecce	Puglia	39.94775N, 18.08637E	52	2015.05	herbaceous vegetation near olive grove	22	7	Yes
Pu2	Gallipoli	Lecce	Puglia	40.05457N, 17.99425E	15	2016.05-08	herbaceous vegetation near olive grove	24	15	Yes
Pu3	Ruvo di Puglia	Bari	Puglia	41.11695N, 16.48882E	230	2016.10	herbaceous vegetation near olive groves	8	8	Yes
Pu4	Fasano	Brindisi	Puglia	40.84022N, 17.36070E	368	2016.05	meadow near olive groves	10	8	Yes
Pu5	Avetrana	Taranto	Puglia	40.35058N, 17.73411E	78	2015.09	olive tree	0	1	No
Pu6	Putignano	Bari	Puglia	40.84707N, 17.08710E	406	2016.04	*Medicago* sp	0	1	No
Pu7	Locorotondo	Bari	Puglia	40.76916N, 17.33388E	365	2016.05	herbaceous vegetation near olive groves	10	1	No
Pu8	Gioia del Colle	Bari	Puglia	40.81237N, 16.92400E	370	2016.06	*Quercus* sp	0	1	No
Pu9	Bari	Bari	Puglia	41.11137N, 17.60473E	21	2015.04	herbaceous vegetation near olive groves	0	1	No
Pu10	Castellaneta	Taranto	Puglia	40.61611N, 16.95888E	180	2018.06	olive trees	0	1	No
Pu11	Alezio	Lecce	Puglia	40.05444N, 18.06138E	62	2015.06	olive trees	0	3	No
Pu12	Galugnano	Lecce	Puglia	40.25643N,	18	2016.05	herbaceous	4	1	No

				18.21200E			vegetation near olive grove			
Pu13	Leverano	Lecce	Puglia	40.30833N, 17.95833E	41	2015.05	herbaceous vegetation near olive grove	10	2	No
Pu14	Matino	Lecce	Puglia	40.03328N, 18.14699E	132	2015.04	herbaceous vegetation near olive groves	0	1	No
Pu15	Nociglia	Lecce	Puglia	40.03888N, 18.31388E	95	2016.05	herbaceous vegetation near olive grove	0	1	No
Pu16	Poggiardo	Lecce	Puglia	40.05262N, 18.37901E	87	2015.05	herbaceous vegetation near olive groves	15	1	No
Pu17	Presicce	Lecce	Puglia	39.90214N, 18.26274E	115	2015.05	herbaceous vegetation near olive groves	0	1	No
Pu18	Racale	Lecce	Puglia	39.95596N, 18.07712E	55	2015.06	olive tree	0	1	No
Pu19	Ruffano	Lecce	Puglia	39.97882N, 18.23254E	152	2015.05	olive trees	0	2	No
Pu20	Surbo	Lecce	Puglia	40.41133N, 18.12942E	36	2015.05	olive trees	0	3	No
Pu21	Ugento	Lecce	Puglia	39.90055N, 18.17111E	93	2016.04	herbaceous vegetation near olive groves	0	1	No
Pu22	Ceglie Messapica	Brindisi	Puglia	40.64569N, 17.52880E	240	2015.05	herbaceous vegetation near olive groves	0	2	No
Pu23	Latiano	Brindisi	Puglia	40.60750N, 17.71694E	108	2015.05	herbaceous vegetation near olive groves	10	2	No
Pu24	Mesagne	Brindisi	Puglia	40.54825N, 17.80286E	73	2015.05	herbaceous vegetation near olive groves	0	1	No
Pu25	Oria	Brindisi	Puglia	40.50250N, 17.60083E	126	2015.05	herbaceous vegetation near olive groves	0	2	No
Pu26	San Pietro V.	Brindisi	Puglia	40.50564N,	40	2015.05	herbaceous	0	1	No

				17.99221E			vegetation near olive groves			
Pu27	San Vito dei N.	Brindisi	Puglia	40.60750N, 17.71694E	108	2015.05	herbaceous vegetation near olive groves	0	2	No
Pu28	Corato	Bari	Puglia	41.08027N, 16.31833E	420	2016.06	cherry tree	0	1	No
Si3	Ragusa	Ragusa	Sicilia	36.91868N, 14.71028E	554	2019.06	meadows	12	13	Yes
