# Supplementary material for: Wolbachia infection and genetic diversity of Italian populations of Philaenus spumarius, the main vector of Xylella fastidiosa in Europe
Source: PLoS One. 2022 Aug 29;17(8):e0272028. doi: 10.1371/journal.pone.0272028 (PMC9423658; doi:10.1371/journal.pone.0272028)
Supplement: S2 Table — (PDF) [file pone.0272028.s011.pdf]

S2 Table. Haplotypes of the *Philaenus spumarius COI* gene and their association with *Wolbachia*.

Population acronym	Location	Region	Haplotype[†]	*Wolbachia* infection status	Nr. individuals	Mitochondrial lineage	GenBank Accession Nr.
**Northern Italy**							
Aa1 (n=8)	Compaccio	Alto Adige	H6	Infected	2	North-eastern	MN812359
			H24	Uninfected	1	Western-Mediterranean	MN812362
			H24	Infected	2	Western-Mediterranean	MN812362
			H98	Infected	1	North-eastern	MN812363
			H102	Infected	2	North-eastern	MN812360
Aa2 (n=13)	Castelrotto	Alto Adige	H24	Infected	1	Western-Mediterranean	MN812366
			H32	Uninfected	3	Western-Mediterranean	MN812367
			H49	Uninfected	1	Eastern-Mediterranean	MN812368
			H95	Infected	4	Western-Mediterranean	MN812369
			H96	Uninfected	1	North-eastern	MN812370
			H97	Infected	1	Western-Mediterranean	MN812371
			H101	Infected	1	Western-Mediterranean	MN812364
			H105	Uninfected	1	Eastern-Mediterranean	MN812365
Aa3 (n=10)	San Michele	Alto Adige	H24	Infected	3	Western-Mediterranean	MN812375
			H24	Uninfected	1	Western-Mediterranean	MN812375
			H95	Infected	2	Western-Mediterranean	MN812376
			H99	Infected	1	North-eastern	MN812377
			H100	Infected	1	North-eastern	MN812372
			H103	Uninfected	1	North-eastern	MN812361
			H104	Infected	1	Western-Mediterranean	MN812373
Li1 (n=2)	Finale Ligure	Liguria	H32	Uninfected	1	Western-Mediterranean	MN812400
			H81	Infected	1	Eastern-Mediterranean	MN812401
Li2 (n=1)	Ortovero	Liguria	H29	Not screened	1	Western-Mediterranean	MW737706
Pi1 (n=12)	Asti	Piemonte	H29	Infected	3	Western-Mediterranean	MN812402
			H32	Infected	3	Western-Mediterranean	MN812403
			H32	Uninfected	4	Western-Mediterranean	MN812403
			H32	Not screened	1	Western-Mediterranean	MN812403

			H127	Infected	1		Western-Mediterranean	MW737781
Pi2_R[‡] (n=1)	Villaromagnano	Piemonte	H32	Not screened	1		Western-Mediterranean	KC111917
Pi3 (n=12)	Venaria	Piemonte	H29	Infected	5		Western-Mediterranean	MW737707
			H32	Uninfected	6		Western-Mediterranean	MW737713
			H125	Uninfected	1		Western-Mediterranean	MW737779
Pi4 (n=1)	Grugliasco	Piemonte	H32	Not screened	1		Western-Mediterranean	MW737714
Pi5 (n=4)	Castellero	Piemonte	H29	Uninfected	1		Western-Mediterranean	MW737708
			H32	Uninfected	2		Western-Mediterranean	MW737715
			H34	Uninfected	1		Western-Mediterranean	MW737725
Pi6 (n=6)	Castellamonte	Piemonte	H29	Uninfected	1		Western-Mediterranean	MW737709
			H32	Infected	2		Western-Mediterranean	MW737716
			H32	Uninfected	1		Western-Mediterranean	MW737716
			H34	Uninfected	1		Western-Mediterranean	MW737726
			H126	Uninfected	1		Western-Mediterranean	MW737780
Pi7 (n=5)	Fossano	Piemonte	H32	Uninfected	4		Western-Mediterranean	MW737717
			H124	Uninfected	1		Western-Mediterranean	MW737776
Ve1 (n=5)	Thiene	Veneto	H32	Uninfected	2		Western-Mediterranean	MN812426
			H34	Uninfected	2		Western-Mediterranean	MN812427
			H49	Uninfected	1		Eastern-Mediterranean	MW737750
Ve2_R[‡] (n=1)	Venezia	Veneto	H47	Not screened	1		Eastern-Mediterranean	KC111932
Ve3 (n=11)	Barbarano V.	Veneto	H32	Uninfected	5		Western-Mediterranean	MW737718
			H34	Uninfected	5		Western-Mediterranean	MW737727
			H78	Uninfected	1		Eastern-Mediterranean	MW737757
Ve4 (n=12)	Bardolino	Veneto	H32	Uninfected	3		Western-Mediterranean	MW737719
			H32	Infected	2		Western-Mediterranean	MW737719
			H34	Uninfected	3		Western-Mediterranean	MW737728
			H34	Not screened	2		Western-Mediterranean	MW737728
			H124	Uninfected	1		Western-Mediterranean	MW737777
			H133	Uninfected	1		Western-Mediterranean	MW737787
Ve5 (n=5)	Belvedere	Veneto	H32	Infected	1		Western-Mediterranean	MW737720
			H32	Uninfected	1		Western-Mediterranean	MW737720
			H34	Uninfected	1		Western-Mediterranean	MW737729

			H128	Uninfected	1	Western-Mediterranean	MW737782
			H129	Uninfected	1	Western-Mediterranean	MW737783
Ve6 (n=11)	Lazise	Veneto	H29	Uninfected	1	Western-Mediterranean	MW737710
			H32	Uninfected	4	Western-Mediterranean	MW737721
			H32	Infected	1	Western-Mediterranean	MW737721
			H49	Uninfected	1	Eastern-Mediterranean	MW737751
			H53	Uninfected	1	Eastern-Mediterranean	MW737753
			H125	Uninfected	1	Western-Mediterranean	MW737778
			H130	Uninfected	1	Eastern-Mediterranean	MW737784
			H131	Uninfected	1	Western-Mediterranean	MW737785
Ve7 (n=6)	Montegrotto T.	Veneto	H32	Uninfected	1	Western-Mediterranean	MW737722
			H34	Uninfected	3	Western-Mediterranean	MW737730
			H49	Uninfected	1	Eastern-Mediterranean	MW737752
			H105	Uninfected	1	Eastern-Mediterranean	MN812374
Ve8 (n=5)	Montecchio P.	Veneto	H32	Uninfected	3	Western-Mediterranean	MW737723
			H34	Uninfected	1	Western-Mediterranean	MW737731
			H106	Infected	1	Western-Mediterranean	MN812425
Ve9 (n=9)	Bussolengo	Veneto	H32	Uninfected	4	Western-Mediterranean	MW737724
			H34	Uninfected	4	Western-Mediterranean	MW737732
			H132	Uninfected	1	Western-Mediterranean	MW737786
Em1_R‡ (n=8)	Parma	Emilia Romagna	H32	Not screened	6	Western-Mediterranean	KC111917
			H33	Not screened	1	Western-Mediterranean	KC111918
			H51	Not screened	1	Eastern-Mediterranean	KC111936
Em2_R‡ (n=1)	Bologna	Emilia-Romagna	H52	Not screened	1	Eastern-Mediterranean	KC111937
**Central Italy**							
Ab1 (n=4)	Colonnella	Abruzzo	H49	Uninfected	2	Eastern-Mediterranean	MN812378
		Abruzzo	H56	Uninfected	1	Eastern-Mediterranean	MN812379
		Abruzzo	H72	Uninfected	1	Eastern-Mediterranean	MN812380
Ab2 (n=6)	Torino di Sangro	Abruzzo	H49	Uninfected	5	Eastern-Mediterranean	MN812381
			H73	Uninfected	1	uncertain position	MN812382
To1_R‡ (n=1)	Firenze	Toscana	H49	Not screened	1	Eastern-Mediterranean	KC111934
To2_R‡ (n=1)	Greve in Chianti	Toscana	H49	Not screened	1	Eastern-Mediterranean	KC111934

To3_R[‡] (n=1)	Livorno	Toscana	H53	Not screened	1	Eastern-Mediterranean	KC111938
La1_R[‡] (n=1)	Roma	Lazio	H53	Not screened	1	Eastern-Mediterranean	KC111938
**Southern Italy**							
Ca1 (n=9)	Montesarchio	Campania	H26	Uninfected	1	Western-Mediterranean	MN812383
			H29	Uninfected	1	Western-Mediterranean	MN812384
			H49	Uninfected	5	Eastern-Mediterranean	MN812385
			H51	Uninfected	1	Eastern-Mediterranean	MN812386
			H75	Uninfected	1	Eastern-Mediterranean	MN812387
Ca2 (n=6)	Giffoni V.P.	Campania	H49	Uninfected	3	Eastern-Mediterranean	MN812388
			H73	Uninfected	1	uncertain position	MN812389
			H76	Uninfected	1	Eastern-Mediterranean	MN812390
			H78	Uninfected	1	Eastern-Mediterranean	MN812391
Ca3 (n=10)	Montevergine	Campania	H49	Uninfected	6	Eastern-Mediterranean	MN812392
			H53	Uninfected	3	Eastern-Mediterranean	MN812393
			H77	Uninfected	1	Eastern-Mediterranean	MN812394
Ca4 (n=6)	Vico Equense	Campania	H49	Uninfected	2	Eastern-Mediterranean	MN812395
			H53	Uninfected	1	Eastern-Mediterranean	MN812396
			H74	Uninfected	1	Eastern-Mediterranean	MN812397
			H79	Uninfected	1	Eastern-Mediterranean	MN812398
			H80	Uninfected	1	Eastern-Mediterranean	MN812399
Ba1 (n=1)	Potenza	Basilicata	H49	Not screened	1	Eastern-Mediterranean	MW737733
Pu1 (n=7)	Alliste	Puglia	H49	Uninfected	2	Eastern-Mediterranean	MN812404
			H83	Uninfected	1	Eastern-Mediterranean	MN812405
			H86	Uninfected	1	Eastern-Mediterranean	MN812406
			H87	Uninfected	1	Eastern-Mediterranean	MN812407
			H91	Uninfected	1	Eastern-Mediterranean	MN812408
			H94	Uninfected	1	Eastern-Mediterranean	MN812409
Pu2 (n=15)	Gallipoli	Puglia	H29	Not screened	1	Western-Mediterranean	MW737711
			H49	Uninfected	3	Eastern-Mediterranean	MN812410
			H49	Not screened	3	Eastern-Mediterranean	MN812410
			H53	Uninfected	3	Eastern-Mediterranean	MN812411
			H85	Uninfected	1	Eastern-Mediterranean	MN812412

			H93	Uninfected	1	Eastern-Mediterranean	MN812413
			H107	Not screened	1	uncertain position	MW737758
			H108	Not screened	1	Eastern-Mediterranean	MW737760
			H109	Not screened	1	Eastern-Mediterranean	MW737761
Pu3 (n=8)	Ruvo di Puglia	Puglia	H49	Not screened	1	Eastern-Mediterranean	MN812414
			H49	Uninfected	2	Eastern-Mediterranean	MN812414
			H78	Uninfected	1	Eastern-Mediterranean	MN812415
			H80	Uninfected	1	Eastern-Mediterranean	MN812416
			H88	Uninfected	1	Eastern-Mediterranean	MN812417
			H89	Uninfected	1	Eastern-Mediterranean	MN812418
			H90	Uninfected	1	Eastern-Mediterranean	MN812419
Pu4 (n=8)	Fasano	Puglia	H49	Not screened	1	Eastern-Mediterranean	MW737734
		Puglia	H53	Uninfected	2	Eastern-Mediterranean	MN812420
		Puglia	H82	Uninfected	1	Eastern-Mediterranean	MN812421
		Puglia	H84	Uninfected	2	Eastern-Mediterranean	MN812422
		Puglia	H85	Uninfected	1	Eastern-Mediterranean	MN812423
		Puglia	H92	Uninfected	1	Eastern-Mediterranean	MN812424
Pu5 (n=1)	Avetrana	Puglia	H49	Not screened	1	Eastern-Mediterranean	MW737735
Pu6 (n=1)	Putignano	Puglia	H122	Not screened	1	Eastern-Mediterranean	MW737774
Pu7 (n=1)	Locorotondo	Puglia	H53	Not screened	1	Eastern-Mediterranean	MW737754
Pu8 (n=1)	Gioia del Colle	Puglia	H49	Not screened	1	Eastern-Mediterranean	MW737736
Pu9 (n=1)	Bari	Puglia	H49	Not screened	1	Eastern-Mediterranean	MW737737
Pu10 (n=1)	Castellaneta	Puglia	H121	Not screened	1	Eastern-Mediterranean	MW737773
Pu11 (n=3)	Alezio	Puglia	H49	Not screened	3	Eastern-Mediterranean	MW737738
Pu12 (n=1)	Galugnano	Puglia	H110	Not screened	1	Eastern-Mediterranean	MW737762
Pu13 (n=2)	Leverano	Puglia	H49	Not screened	2	Eastern-Mediterranean	MW737739
Pu14 (n=1)	Matino	Puglia	H49	Not screened	1	Eastern-Mediterranean	MW737740
Pu15 (n=1)	Nociglia	Puglia	H49	Not screened	1	Eastern-Mediterranean	MW737741
Pu16 (n=1)	Poggiardo	Puglia	H49	Not screened	1	Eastern-Mediterranean	MW737742
Pu17 (n=1)	Presicce	Puglia	H49	Not screened	1	Eastern-Mediterranean	MW737743
Pu18 (n=1)	Racale	Puglia	H49	Not screened	1	Eastern-Mediterranean	MW737744
Pu19 (n=2)	Ruffano	Puglia	H111	Not screened	1	Eastern-Mediterranean	MW737763

			H112	Not screened	1	Eastern-Mediterranean	MW737764
Pu20 (n=3)	Surbo	Puglia	H49	Not screened	2	Eastern-Mediterranean	MW737745
			H113	Not screened	1	Eastern-Mediterranean	MW737765
Pu21 (n=1)	Ugento	Puglia	H114	Not screened	1	Eastern-Mediterranean	MW737766
Pu22 (n=2)	Ceglie M.	Puglia	H49	Not screened	1	Eastern-Mediterranean	MW737746
			H115	Not screened	1	Eastern-Mediterranean	MW737767
Pu23 (n=2)	Latiano	Puglia	H53	Not screened	1	Eastern-Mediterranean	MW737755
			H116	Not screened	1	Eastern-Mediterranean	MW737768
Pu24 (n=1)	Masagne	Puglia	H117	Not screened	1	Eastern-Mediterranean	MW737769
Pu25 (n=2)	Oria	Puglia	H49	Not screened	1	Eastern-Mediterranean	MW737747
			H118	Not screened	1	Eastern-Mediterranean	MW737770
Pu26 (n=1)	S. Pietro V.	Puglia	H119	Not screened	1	Eastern-Mediterranean	MW737771
Pu27 (n=1)	S. Vito N.	Puglia	H49	Not screened	1	Eastern-Mediterranean	MW737748
Pu27 (n=1)	S. Vito N.	Puglia	H120	Not screened	1	Eastern-Mediterranean	MW737772
Pu28 (n=1)	Corato	Puglia	H53	Not screened	1	Eastern-Mediterranean	MW737756
Si1_R[‡] (n=1)	Aci Sant'Antonio	Sicilia	H49	Not screened	1	Eastern-Mediterranean	KC111934
Si2_R[‡] (n=1)	Tarderia	Sicilia	H29	Not screened	1	Western-Mediterranean	KC111914
Si3 (n=13)	Ragusa	Sicilia	H29	Not screened	1	Western-Mediterranean	MW737712
			H29	Uninfected	9	Western-Mediterranean	MW737712
			H49	Uninfected	1	Eastern-Mediterranean	MW737749
			H107	Uninfected	1	uncertain position	MW737759
			H123	Uninfected	1	Western-Mediterranean	MW737775

[†]Haplotypes have been named following the progressive numbering by [46] and the new haplotypes from our study start from H72.

[‡]Individuals sequenced by [46].
