# Supplementary material for: Wolbachia infection and genetic diversity of Italian populations of Philaenus spumarius, the main vector of Xylella fastidiosa in Europe
Source: PLoS One. 2022 Aug 29;17(8):e0272028. doi: 10.1371/journal.pone.0272028 (PMC9423658; doi:10.1371/journal.pone.0272028)
Supplement: S3 Table — Sex ratio in the populations of Philaenus spumarius (A) and statistical analysis (G-test of independence) (B). (PDF) [file pone.0272028.s012.pdf]

S3 Table. Sex ratio in the populations of *Philaenus spumarius* (A) and statistical analysis (G-test of independence) (B).

(A)

Region	Population	*Wolbachia* infection rate[†]	N. of adults collected in the field		Proportion of females
			Females	Males	
**NORTHERN ITALY**			246	168	59%
**Alto Adige**	Aa1	76.60%	43	27	61.43%
	Aa2	27.59%	56	50	52.83%
	Aa3	80%	45	28	61.64%
	Total	*57.93%*	*144*	*105*	*57.83%*
**Piemonte**	Pi1	53.33%	69	51	57.50%
**Liguria**	Li1	25%	33	12	73.33%
**SOUTHERN ITALY**			525	429	55.03%
**Campania**	Ca1	0	10	7	58.82%
	Ca2	0	9	12	42.86
	Ca3	0	8	9	47.06
	Ca4	0	20	30	40%
	Ca5	0	27	12	69.23%
	Ca6	0	21	18	53.85%
	Ca8	0	27	23	54%
	Ca9	0	8	9	47.06%
	Ca13	0	9	18	33.33%
	Ca14	0	30	24	55.55%
	Ca15	0	12	7	63.16%
	Ca16	0	11	16	40.74%
	Ca17	0	10	8	55.55%
	Ca18	0	9	4	69.23%
	Ca19	0	19	8	70.37%
	Ca20	0	29	26	52.73%
	Total	*0*	*259*	*231*	*52.86%*
**Puglia**	Pu1	0	26	13	66.67%
	Pu2	0	42	85	33.07%
	Pu3	0	27	14	65.85%
	Pu4	0	16	17	48.48%
	Pu7	0	22	3	88%
	Pu12	0	70	18	79.54%
	Pu13	0	15	16	48.39%
	Pu16	0	12	12	50%
	Pu23	0	29	14	67.44%
	Total	*0*	*259*	*192*	*57.43%*
**Sicilia**	Si3	0	7	6	53.85%

(B)

Comparisons	G-value	d.f.	P-value
Between populations of Alto Adige	1.89	2	0.39
Between populations of northern Italy	6.14	4	0.19
Between populations of Campania	20.93	15	0.14
Between populations of Puglia	68.01	8	1.22E-11
Between populations of southern Italy	90.93	25	2.01E-09
Between regions of Italy	8.51	5	0.13
Between regions of northern Italy	4.25	2	0.12
Between regions of southern Italy	1.99	2	0.37
Between northern and southern Italy	2.27	1	0.13
