# Supplementary material for: Wolbachia infection and genetic diversity of Italian populations of Philaenus spumarius, the main vector of Xylella fastidiosa in Europe
Source: PLoS One. 2022 Aug 29;17(8):e0272028. doi: 10.1371/journal.pone.0272028 (PMC9423658; doi:10.1371/journal.pone.0272028)
Supplement: S4 Table — n, number of haplotypes; S, segregating sites; Hd, haplotype diversity; π, nucleotide diversity; k, mean number of pairwise differences; w, group including Wolbachia-infected populations; NE, without haplotypes of the north-eastern lineage. (PDF) [file pone.0272028.s013.pdf]

S4 Table. Molecular diversity parameters of *Philaenus spumarius* and neutrality tests for the overall data set and the populations grouped by region of origin. *n*, number of haplotypes; *S*, segregating sites; *Hd*, haplotype diversity; π, nucleotide diversity; *k*, mean number of pairwise differences; [w], group including *Wolbachia*-infected populations; [NE], without haplotypes of the north-eastern lineage.

Geographic groups	Molecular diversity parameters					Neutrality Test	
(n° of individuals)	*n*	*S*	*Hd*	*π*	*k*	Tajima's D	Fu's FS
Total (226)	58	69	0.891	0.006	3.214	-2.168 (P<0.01)	-26.064 (P<0.01)
Northern Italy (134) [w]	30	35	0.805	0.005	2.808	-1.679 (P=0.01)	-17.202 (P<0.01)
Alto Adige (31) [w]	15	24	0.896	0.010	5.303	-0.413 (P=0.36)	-2.860 (P=0.14)
Alto Adige (23) [NE, w]	9	13	0.818	0.005	2.862	-0.657(P=0.27)	-1.519 (P=0.22)
Piemonte (39) [w]	7	6	0.596	0.001	0.742	-1.280 (P=0.09)	-3.436 (P<0.01)
Veneto (64) [w]	16	15	0.720	0.003	1.745	-1.327 (P=0.09)	-8.528 (P<0.01)
South-Central Italy (92)	32	46	0.824	0.004	2.092	-2.449 (P<0.01)	-27.289 (P<0.01)
Abruzzo (10)	4	4	0.533	0.001	0.8	-1.667 (P=0.02)	-1.346 (P=0.03)
Campania (31)	13	15	0.729	0.003	1.432	-2.070 (P<0.01)	-9.290 (P<0.01)
Puglia (38)	21	37	0.889	0.005	2.667	-2.457 (P<0.01)	-15.79 (P<0.01)
Sicilia (13)	4	3	0.423	0.001	0.590	-1.233 (P=0.10)	-1.658 (P=0.02)
