# Supplementary material for: Wolbachia infection and genetic diversity of Italian populations of Philaenus spumarius, the main vector of Xylella fastidiosa in Europe
Source: PLoS One. 2022 Aug 29;17(8):e0272028. doi: 10.1371/journal.pone.0272028 (PMC9423658; doi:10.1371/journal.pone.0272028)
Supplement: S5 Table — n, number of haplotypes; S, segregating sites; Hd, haplotype diversity; π, nucleotide diversity; k, mean number of pairwise differences. (PDF) [file pone.0272028.s014.pdf]

S5 Table. Molecular diversity parameters and neutrality tests for the haplotypes of *Philaenus spumarius* grouped by mitochondrial lineage. *n*, number of haplotypes; *S*, segregating sites; *Hd*, haplotype diversity; *π*, nucleotide diversity; *k*, mean number of pairwise differences.

Geographic groups†	Molecular diversity parameters					Neutrality Test	
Mitochondrial lineages†	***n***	***S***	***Hd***	***π***	***k***	**Tajima's D**	**Fu's FS**
**ITALY (222)**	56	69	0.887	0.006	3.248	-2.165 (P<0.01)	-26.064 (P<0.01)
Eastern-Mediterranean (83)	29	46	0.755	0.003	1.737	-2.617 (P<0.01)	-27.537 (P<0.01)
Western-Mediterranean (130)	20	21	0.770	0.003	1.727	-1.561 (P=0.03)	-10.831 (P<0.01)
North-eastern (9)	7	14	0.944	0.007	3.722	-1.331 (P=0.09)	-1.960 (P=0.08)
**NORTHERN ITALY (132)**	30	35	0.807	0.005	2.831	-1.675 (P=0.025)	-17.182 (P<0.001)
Eastern-Mediterranean (9)	5	4	0.806	0.002	1.056	-1.149 (P=0.137)	-2.360 (P=0.008)
Western-Mediterranean (114)	18	20	0.743	0.003	1.829	-1.466 (P=0.040)	-8.187 (P=0.006)
North-eastern (9)	7	14	0.944	0.007	3.722	-1.331 (P=0.09)	-1.960 (P=0.08)
**ALTO ADIGE (31)**	15	24	0.897	0.010	5.303	-0.413 (P=0.387)	-2.860 (P=0.149)
North-eastern (9)	7	14	0.944	0.007	3.722	-1.331 (P=0.09)	-1.960 (P=0.08)
Western-Mediterranean (20)	6	9	0.758	0.004	2.089	-0.606 (P=0.298)	-0.147 (P=0.486)
**PIEMONTE (39)**	7	6	0.596	0.001	0.742	-1.280 (P=0.09)	-3.436 (P=0.009)
Western-Mediterranean (38)	7	6	0.606	0.001	0.757	-1.269 (P=0.105)	-3.401 (P=0.013)
**VENETO (63)**	16	15	0.721	0.003	1.750	-1.331 (P=0.081)	-8.573 (P=0.002)
Western-Mediterranean (56)	11	10	0.649	0.002	1.264	-1.175 (P=0.121)	-5.004 (P=0.010)
Eastern-Mediterranean (7)	5	4	0.857	0.002	1.143	-1.434 (P=0.052)	-2.858 (P=0.003)
**SICILIA (13)**							
Western-Mediterranean (11)	2	1	0.200	0.0001	0.200	-1.112(P=0.200)	-0.339 (P=1.147)

†N° of individuals in brackets
