# Supplementary material for: Wolbachia infection and genetic diversity of Italian populations of Philaenus spumarius, the main vector of Xylella fastidiosa in Europe
Source: PLoS One. 2022 Aug 29;17(8):e0272028. doi: 10.1371/journal.pone.0272028 (PMC9423658; doi:10.1371/journal.pone.0272028)
Supplement: S6 Table — n, number of haplotypes; S, segregating sites; Hd, haplotype diversity; π, nucleotide diversity; k, mean number of pairwise differences. (PDF) [file pone.0272028.s015.pdf]

S6 Table. Molecular diversity parameters and neutrality tests for the individuals of *Philaenus spumarius* grouped by the status of *Wolbachia* infection. *n,* number of haplotypes; *S*, segregating sites; *Hd*, haplotype diversity; *π*, nucleotide diversity; *k*, mean number of pairwise differences.

Groups	Molecular diversity parameters					Neutrality Test	
(n° of individuals)	*n*	*S*	*Hd*	*π*	*k*	Tajima's D	Fu's FS
**Italy (214)**	56	68	0.892	0.006	1.692	-2.155 (P<0.01)	-25.995 (P<0.01)
Infected (41)	14	18	0.883	0.007	4.041	-0.128 (P=0.49)	-2.278 (P= 0.18)
Uninfected (173)	45	62	0.875	0.005	2.779	-2.273 (P<0.01)	-26.44 (P<0.01)
**Northern Italy (132)**	30	35	0.807	0.005	2.831	-1.675 (P=0.025)	-17.182 (P<0.001)
Infected (41)	14	18	0.883	0.007	4.041	-0.128 (P=0.520)	-2.278 (P=0.187)
Uninfected (91)	19	25	0.720	0.004	1.927	-1.834 (P=0.017)	-9.890 (P=0.001)
**Alto Adige (31)**	15	24	0.897	0.010	5.303	-0.413 (P=0.387)	-2.860 (P=0.149)
Infected (22)	10	15	0.861	0.009	5.004	0.779 (P=0.809)	-0.526 (P=0.415)
Uninfected (9)	6	16	0.889	0.009	4.833	-0.868 (P=0.218)	-0.078 (P=0.466)
**Piemonte (38)**	7	6	0.606	0.001	0.757	-1.269 (P=0.970)	-3.401 (P=0.008)
Infected (14)	3	2	0.582	0.001	0.670	0.179 (P=0.658)	0.055 (P=0.397)
Uninfected (24)	6	5	0.500	0.001	0.699	-1.384 (P=0.073)	-3.109 (P=0.003)
**Veneto (63)**	16	15	0.721	0.003	1.750	-1.331 (P=0.081)	-8.573 (P=0.002)
Infected (5)	2	1	0.400	0.001	0.400	-0.816 (P=0.311)	0.090 (P=0.298)
Uninfected (58)	15	14	0.730	0.003	1.797	-1.202 (P=0.104)	-7.359 (P=0.002)
**Italy**							
**Western-Mediterranean (126)**	20	21	0.770	0.003	1.752	-1.556 (P=0.030)	-10.800 (P=0.001)
Infected (34)	9	11	0.832	0.004	2.419	-0.316 (P=0.419)	-1.182 (P=0.043)
Uninfected (92)	14	14	0.710	0.002	1.235	-1.523 (P=0.312)	-7.553 (P=0.002)
**Northern Italy**							
**Western-Mediterranean (114)**	18	20	0.743	0.003	1.829	-1.466 (P=0.040)	-8.187 (P=0.006)
Infected (34)	9	11	0.832	0.004	2.419	-0.316 (P=0.413)	-1.182 (P=0.302)
Uninfected (80)	12	12	0.639	0.002	1.241	-1.338 (P=0.072)	-5.407 (P=0.180)
**Eastern-Mediterranean (9)**							
Uninfected (9)	5	4	0.806	0.002	1.056	-1.149 (P=0.137)	-2.360 (P=0.008)
**Alto Adige**							
**Western-Mediterranean (20)**	6	9	0.758	0.004	2.089	-0.606 (P=0.298)	-0.147 (P=0.486)
Infected (15)	5	6	0.714	0.003	1.562	-0.538 (P=0.312)	-0.416 (P=0.973)
Uninfected (5)	2	3	0.600	0.003	1.800	1.573 (P=0.402)	2.429 (P=0.854)
**North-eastern (9)**	7	14	0.944	0.007	3.722	-1.331 (P=0.09)	-1.960 (P=0.08)
Infected (7)	5	6	0.905	0.004	2.000	-0.931(P=0.244)	-1.548 (P=0.078)
Uninfected (2)	2	8	1.000	0.015	8.000	0.000 (P=1.000)	2.079 (P=0.569)
**Piemonte**							
**Western-Mediterranean (38)**	**7**	**6**	**0.606**	**0.001**	**0.757**	**-1.269 (P=0.105)**	**-3.401 (P=0.013)**
Infected (14)	3	2	0.582	0.001	0.670	0.179 (P=0.623)	0.055 (P=0.074)
Uninfected (24)	6	5	0.500	0.001	0.699	-1.384 (P=0.394)	-3.109 (P=0.003)
**Veneto**							

**Western-Mediterranean (56)**	11	10	0.649	0.002	1.264	-1.175 (P=0.121)	-5.004 (P=0.010)
Infected (5)	2	1	0.400	0.001	0.400	-0.816 (P=0.303)	0.090 (P=0.151)
Uninfected (51)	10	9	0.653	0.002	1.280	-1.004 (P=0.277)	-4.003 (P=0.014)
**Eastern-Mediterranean (9)**							
Uninfected (9)	5	4	0.806	0.002	1.056	-1.149 (P=0.137)	-2.360 (P=0.008)
