# Supplementary material for: Wolbachia infection and genetic diversity of Italian populations of Philaenus spumarius, the main vector of Xylella fastidiosa in Europe
Source: PLoS One. 2022 Aug 29;17(8):e0272028. doi: 10.1371/journal.pone.0272028 (PMC9423658; doi:10.1371/journal.pone.0272028)
Supplement: S7 Table — (PDF) [file pone.0272028.s016.pdf]

S7 Table. Analysis of molecular variance (AMOVA) based on *COI* gene sequences of 27 populations of *Philaenus spumarius.*

Grouping	Source of variation	df	Variation (%)	Interpopulation fixation indices	
				F_{CT}	F_{ST}
Geography – populations grouped by Italian regions (Alto Adige, Abruzzo, Campania, Piemonte, Puglia, Sicilia, Veneto)	Among groups	6	39.16	0.392	0.430
	Within groups	20	3.82	(P<0.001)	(P<0.001)
	Within populations	199	57.02		
Geography – populations grouped by Italian macro-areas (Northern Italy, South-Central Italy)	Among groups	1	35.18	0.352	0.501
	Within groups	25	14.91	(P<0.001)	(P<0.001)
	Within populations	199	49.91		
Wolbachia infection status – populations grouped in infected and uninfected	Among groups	1	20.15	0.201	0.455
	Within groups	25	25.40	(P<0.001)	(P<0.001)
	Within populations	199	54.45		
