# Supplementary material for: Wolbachia infection and genetic diversity of Italian populations of Philaenus spumarius, the main vector of Xylella fastidiosa in Europe
Source: PLoS One. 2022 Aug 29;17(8):e0272028. doi: 10.1371/journal.pone.0272028 (PMC9423658; doi:10.1371/journal.pone.0272028)
Supplement: S8 Table — (PDF) [file pone.0272028.s017.pdf]

S8 Table. Analysis of molecular variance (AMOVA) run on *COI* gene haplotypes of *Philaenus spumarius* individuals grouped according their infection status (infected or uninfected).

Geographic area	Source of variation	df	Variation (%)	F_{ST}
Northern Italy	Among groups	1	18.53	0.185 (P<0.001)
	Within groups	130	81.48	
Alto Adige	Among groups	1	14.09	0.140 (P=0.023)
	Within groups	29	85.91	
Piemonte	Among groups	1	17.11	0.171 (P=0.007)
	Within groups	36	82.89	
Veneto	Among groups	1	14.92	0.149 (P=0.045)
	Within groups	61	85.08	
