# Supplementary material for: Wolbachia infection and genetic diversity of Italian populations of Philaenus spumarius, the main vector of Xylella fastidiosa in Europe
Source: PLoS One. 2022 Aug 29;17(8):e0272028. doi: 10.1371/journal.pone.0272028 (PMC9423658; doi:10.1371/journal.pone.0272028)
Supplement: S9 Table — (PDF) [file pone.0272028.s018.pdf]

S9 Table. Analysis of molecular variance (AMOVA) run on *COI* gene haplotypes of individuals of the western-Mediterranean lineage of *Philaenus spumarius* grouped according their infection status (infected or uninfected).

Geographic area	Source of variation	df	Variation (%)	F_{ST}
Northern Italy	Among groups	1	26.42	0.265 (P<0.001)
	Within groups	112	73.54	
Alto Adige	Among groups	1	42.68	0.426 (P=0.003)
	Within groups	18	57.32	
Piemonte	Among groups	1	17.11	0.171 (P=0.008)
	Within groups	36	82.89	
Veneto	Among groups	1	19.74	0.197 (P=0.049)
	Within groups	54	80.26	
