# Supplementary material for: Wolbachia infection and genetic diversity of Italian populations of Philaenus spumarius, the main vector of Xylella fastidiosa in Europe
Source: PLoS One. 2022 Aug 29;17(8):e0272028. doi: 10.1371/journal.pone.0272028 (PMC9423658; doi:10.1371/journal.pone.0272028)
Supplement: S10 Table — (PDF) [file pone.0272028.s019.pdf]

S10 Table. *Wolbachia* sequence types (ST) of *Philaenus spumarius* and their association with host population and haplotype.

Population	Location	Region	COI haplotype	Mitochondrial lineage†	*Wolbachia* ST	*wsp* allele	GenBank Accession Nr					
							gatB	***coxA***	***hcpA***	***ftsZ***	***fbpA***	***wsp***
Aa1	Compaccio	Alto Adige	H6	NE	546	698	MN812316	MN812292	MN812328	MN812340	MN812304	MN812352
Aa1	Compaccio	Alto Adige	H98	NE	545	698	MN812317	MN812293	MN812329	MN812341	MN812305	MN812352
Aa1	Compaccio	Alto Adige	H6	NE	539	698	MN812318	MN812294	MN812330	MN812342	MN812306	MN812352
Aa1	Compaccio	Alto Adige	H102	NE	537	n.d.	MN812319	MN812295	MN812331	MN812343	MN812307	MN812353
Aa2	Castelrotto	Alto Adige	H95	W-Med	538	698	MN812320	MN812296	MN812332	MN812344	MN812308	MN812354
Aa2	Castelrotto	Alto Adige	H97	W-Med	539	698	MN812321	MN812297	MN812333	MN812345	MN812309	MN812354
Aa2	Castelrotto	Alto Adige	H95	W-Med	538	698	MN812320	MN812296	MN812332	MN812344	MN812308	MN812354
Aa2	Castelrotto	Alto Adige	H101	W-Med	539	698	MN812321	MN812297	MN812333	MN812345	MN812309	MN812354
Aa3	San Michele	Alto Adige	H24	W-Med	539	698	MN812322	MN812298	MN812334	MN812346	MN812310	MN812355
Aa3	San Michele	Alto Adige	H99	NE	538	698	MN812323	MN812299	MN812335	MN812347	MN812311	MN812355
Aa3	San Michele	Alto Adige	H24	W-Med	539	698	MN812322	MN812298	MN812334	MN812346	MN812310	MN812355
Aa3	San Michele	Alto Adige	H95	W-Med	538	698	MN812323	MN812299	MN812335	MN812347	MN812311	MN812355
Aa3	San Michele	Alto Adige	H100	NE	539	698	MN812322	MN812298	MN812334	MN812346	MN812310	MN812355
Aa3	San Michele	Alto Adige	H24	W-Med	539	698	MN812322	MN812298	MN812334	MN812346	MN812310	MN812355
Aa3	San Michele	Alto Adige	H104	W-Med	539	698	MN812322	MN812298	MN812334	MN812346	MN812310	MN812355
Li1	Finale Ligure	Liguria	H81	E-Med	549	698	MN812324	MN812300	MN812336	MN812348	MN812312	MN812356
Pi1	Asti	Piemonte	H29	W-Med	539	698	MN812325	MN812301	MN812337	MN812349	MN812313	MN812357
Pi1	Asti	Piemonte	H32	W-Med	549	698	MN812326	MN812302	MN812338	MN812350	MN812314	MN812357
Pi6	Castellamonte	Piemonte	H32	W-Med	549	698	MN812326	MN812302	MN812338	MN812350	MN812314	MN812357
Ve8	Montecchio P.	Veneto	H106	W-Med	549	698	MN812327	MN812303	MN812339	MN812351	MN812315	MN812358

† E-Med = eastern-Mediterranean lineage; NE = eastern lineage; W-Med = western-Mediterranean lineage
