# Supplementary material for: Wolbachia infection and genetic diversity of Italian populations of Philaenus spumarius, the main vector of Xylella fastidiosa in Europe
Source: PLoS One. 2022 Aug 29;17(8):e0272028. doi: 10.1371/journal.pone.0272028 (PMC9423658; doi:10.1371/journal.pone.0272028)
Supplement: S11 Table — (PDF) [file pone.0272028.s020.pdf]

S11 Table. Distribution of mitochondrial lineages of *Philaenus spumarius* among the geographic regions of Italy

Geographic area	N° of individuals	Mitochondrial lineages			
		Eastern-Mediterranean	Western-Mediterranean	North-eastern	Uncertain position
**SOUTHERN ITALY**					
Basilicata	1	1	0	0	0
Campania	31	28	2	0	1
Puglia	72	70	1	0	1
Sicilia†	15	2	12	0	1
Total	*119*	*101*	*15*	*0*	*3*
**CENTRAL ITALY**					
Abruzzo	10	9	0	0	1
Lazio‡	1	1	0	0	0
Toscana‡	3	3	0	0	0
Total	*14*	*13*	*0*	*0*	*1*
**NORTHERN ITALY**					
Alto Adige	31	2	20	9	0
Emilia-Romagna‡	9	2	7	0	0
Liguria	3	1	2	0	0
Piemonte	41	0	41	0	0
Veneto	65	8	57	0	0
Total	*149*	*13*	*127*	*9*	*0*

†Two individuals were from [46] (see Table S2).
‡All individuals were from [46] (see Table S2).
